# ‘Atherothrombosis-associated microRNAs in Antiphospholipid syndrome and Systemic Lupus Erythematosus patients’

**DOI:** 10.1038/srep31375

**Published:** 2016-08-09

**Authors:** C. Pérez-Sánchez, M. A. Aguirre, P. Ruiz-Limón, N. Barbarroja, Y. Jiménez-Gómez, I. Arias de la Rosa, A. Rodriguez-Ariza, E. Collantes-Estévez, P. Segui, F. Velasco, M. J. Cuadrado, R. Teruel, R. González-Conejero, C. Martínez, Ch. López-Pedrera

**Affiliations:** 1 Maimonides Institute for Research in Biomedicine of Cordoba (IMIBIC)/Reina Sofia University Hospital/University of Cordoba, Cordoba, Spain; 2 Lupus Research Unit, St Thomas Hospital, London, United Kingdom; 3 Regional Centre for Blood Donation, University of Murcia, IMIB-Arrixaca, Spain

## Abstract

MicroRNAs markedly affect the immune system, and have a relevant role in CVD and autoimmune diseases. Yet, no study has analyzed their involvement in atherothrombosis related to APS and SLE patients. This study intended to: 1) identify and characterize microRNAs linked to CVD in APS and SLE; 2) assess the effects of specific autoantibodies. Six microRNAs, involved in atherothrombosis development, were quantified in purified leukocytes from 23 APS and 64 SLE patients, and 56 healthy donors. Levels of microRNAs in neutrophils were lower in APS and SLE than in healthy donors. Gene and protein expression of miRNA biogenesis-related molecules were also reduced. Accordingly, more than 75% of identified miRNAs by miRNA profiling were underexpressed. In monocytes, miR124a and -125a were low, while miR-146a and miR-155 appeared elevated. Altered microRNAs’ expression was linked to autoimmunity, thrombosis, early atherosclerosis, and oxidative stress in both pathologies. *In vitro* treatment of neutrophils, monocytes, and ECs with aPL-IgG or anti-dsDNA-IgG antibodies deregulated microRNAs expression, and decreased miRNA biogenesis-related proteins. Monocyte transfections with pre-miR-124a and/or -125a caused reduction in atherothrombosis-related target molecules. In conclusion, microRNA biogenesis, significantly altered in neutrophils of APS and SLE patients, is associated to their atherothrombotic status, further modulated by specific autoantibodies.

Accumulating evidence shows that humoral autoimmunity might play a relevant role in cardiovascular disease (CVD). Some autoantibodies, present in patients with antiphospholipid syndrome (APS) and systemic lupus erythematosus (SLE), possibly represent emerging cardiovascular (CV) risk factors. Thus, previous studies have demonstrated that antiphospholipid antibodies (aPL) provoke a pro-atherothrombotic state through the induced expression of both prothrombotic and proinflammatory molecules, as well as through the induction of oxidative stress and mitochondrial dysfunction in monocytes and neutrophils^1^,^2^,^3^. Furthermore, studies have shown that endothelial cells (EC) expressed significantly higher amounts of adhesion molecules (ICAM-1, VCAM-1 and E-selectin) when incubated with aPL antibodies and ß2GP1 *in vitro*^4^,^5^. Similarly, the incubation of ECs with antibodies reacting with ß2GP1 has been shown to induce EC activation with upregulation of Tissue factor (TF)^6^, adhesion molecules, IL-6 production and alteration in prostaglandin metabolism^5^.

On the other hand, the immunologic hallmarks of lupus are autoantibodies against nuclear proteins and anti-double-stranded DNA, so that anti-dsDNA titres correlate with disease activity and are associated with specific tissue damage^7^,^8^. In addition, anti-dsDNA antibodies titres are linked to the expression of inflammatory mediators (in plasma and immune cells) that characterize that autoimmune condition^9^,^10^. Moreover, *in vitro* treatment of ECs with anti-DNA autoantibodies has been shown to upregulate IL-1, IL-6, IL-8, transforming growth factor beta, nitric oxide synthase, and adhesion molecules expression, thus providing evidence that anti-dsDNA could play an important pathogenic role in inducing inflammatory injury of vascular endothelium in SLE^11^,^12^,^13^.

Pathophysiological mechanisms connecting atherosclerosis and CVD with APS and SLE have been greatly broadened with the application of genomic technologies, which have allowed explaining how these alterations might be associated to each autoimmune disease^14^,^15^,^16^. One emerging and important mechanism controlling gene expression is epigenetics, which controls gene packaging and expression independent of alterations in the DNA sequence. Epigenetics, which comprises DNA methylation, histone modifications, and microRNA (miRNA) activity, is providing new directions linking genomics and environmental factors^17^.

MicroRNAs are small noncoding RNAs of approximately 19–25 nucleotides in length^18^ originated as pri- and pre-miRNAs, and processed by different ribonucleases such as Drosha and Dicer. miRNAs are ubiquitously expressed in a wide range of species and negatively regulate gene expression at the post-transcriptional level by targeting specific mRNAs for degradation or suppressing mRNA translation^18^,^19^. The whole human genome is estimated to encode 2588 mature miRNAs (miRbase v21, June 2014), which are predicted to target one third of human genes^20^.

A number of works have analyzed the expression profile of miRNAs in peripheral blood cells, biological fluids, and tissues of patients with SLE. These works have shown that differential expression of multiple miRNAs seems to contribute to SLE pathogenesis, by regulating the type I interferon pathway, inflammatory cytokine expression, DNA methylation in T cells and local tissue inflammation (i.e. miR-15, miR-21, miR-31, miR-125a, miR142, miR-146a, miR-155, and miR-181, among others)^21^. Moreover, the expression of deregulated miRNAs in SLE patients influences some parameters of the disease activity and severity^20^,^22^. In APS, miRNAs from miR-17-92 cluster were identified as potential modulators of the expression of TF, the main inductor of thrombosis in APS patients^23^.

Some studies have highlighted the role of miRNAs in processes such as oxidative stress and CVD, including atherosclerosis^24^. However, no study has identified and characterized miRNAs associated with CV and atherothrombotic risks observed in APS and SLE.

Thus, we undertake this work: 1) to identify and to characterize miRNAs related to the pathogenesis of CVD in APS and SLE patients. 2) to assess the effects of specific autoantibodies in the regulation of those epigenetic processes.

## Results

### Bioinformatic prediction of miRNAs having specific targets related to thrombosis and inflammation and quantification in leukocyte subsets

**Figure 1:**
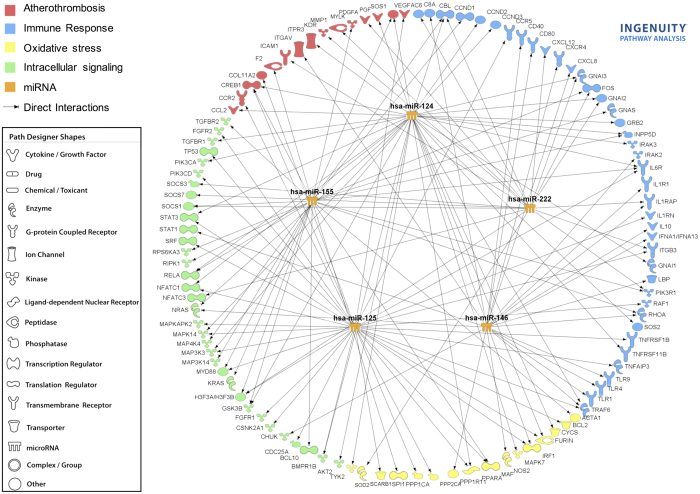
Interaction Network of miRNAs and mRNA target involved in cardiovascular disease (atherothrombosis, immune response, oxidative stress and intracellular signaling). By using the tool microRNA Target Filter of QIAGEN’s Ingenuity Pathway Analysis (IPA^®^, QIAGEN Redwood City, www.qiagen.com/ingenuity), the software generated a network including the selected miRNAs and their mRNA targets, filtered by connective tissue disorders. Only targets experimentally observed and predicted with high confidence are shown and related by direct interactions with their specific miRNA regulators.

*In silico* studies were performed in order to determine the possible regulation of prothrombotic and proinflammatory molecules by miRNAS. We used the web-tool miRo (http://ferrolab.dmi.unict.it/miro)^25^, to allow the simultaneous visualization from the results of three different restrictive algorithms Targetscan (http://www.targetscan.org), PicTar (http://pictar.bio.nyu.edu), and miRanda (http://microrna.sanger.ac.uk). Indeed, our research focused on those miRNAs that were predicted to repress those proinflammatory molecules by the three algorithms. The most relevant miRNAs identified following this method were miR-124a-3p, miR-125a-5p, miR-125b-5p, miR-146a-5p, miR-155-5p, and miR-222-3p. Potential mRNA targets involved in processes such as atherothrombosis, immune response, oxidative stress and intracellular signaling were identified through the use of QIAGEN’s Ingenuity^®^ Pathway Analysis (IPA^®^, QIAGEN Redwood City, www.qiagen.com/ingenuity). We further developed a network that integrates the interaction miRNA-mRNA target (Fig. 1).

**Figure 2:**
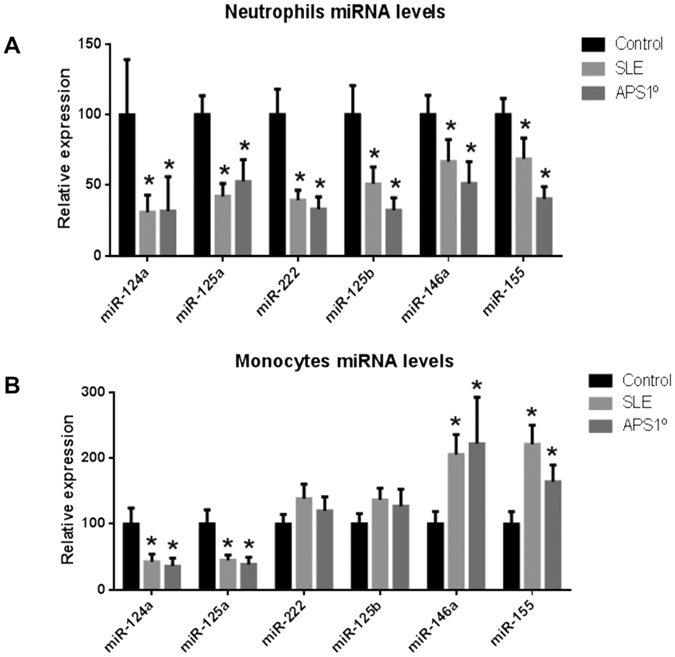
Expression levels of the selected miRNAs in monocytes and neutrophils from APS and SLE patients. miRNAs levels were measured in all the subjects included in the study (23 APS, 64 SLE, and 56 healthy donors) on isolated neutrophils (**A**) or monocytes (**B**) by qRT-PCR and normalized with U6 snRNA. Differences were analyzed by means of Student’s t test. Statistical significance was taken as p < 0.05.

Analyses performed on both APS and SLE patients showed that the expression levels of all the selected miRNAs in neutrophils were found significantly decreased in relation to the control group (Fig. 2A). Mir124a and miR-125a were also found reduced in monocytes from APS and SLE patients, while miR-146a and miR-155 appeared significantly increased (Fig. 2B). No significant changes in the expression of miRNAs were found in lymphocytes from APS or SLE patients (data not shown).

### Potential influence of therapy on miRNA modulation in APS and SLE patients

Numerous studies reported that miRNAs’ expression can be modulated by effect of specific therapies^26^. To assess this aspect in our cohort of patients, we classified them in three groups that could discriminate the treatments received, including primary APS patients, SLE patients negative for aPL, and SLE patients positive for aPL. Then, we distributed them on different groups of treatment, on which we performed statistical analyses.

On primary APS patients (n = 23), we identified 17 patients that had been treated only with oral anti-platelets/anticoagulants (A/A), 3 treated with A/A plus antimalarials (hydroxychloroquine, HCQ), two patients that received A/A +HCQ +prednisone, and 1 patient with no drug treatment (Supplemental Table IV). Comparison between patients treated with A/A vs those treated with A/A +HCQ showed no significant differences on miRNA expression (data not shown).

On SLE patients positive for aPL (n = 23), we found 12 patients that received combined treatment with A/A, HCQ, and prednisone; 5 patients treated with A/A plus prednisone; 3 patients taking A/A +HCQ + Prednisone + Immunosuppressive drugs; 2 patients treated with A/A; and one patient treated with A/A +HCQ (Supplemental Table V). Statistical analyses between groups of patients receiving AA +HCQ+prednisone vs patients treated with AA +prednisone demonstrated no significant differences on miRNA expression (data not shown).

Finally, on SLE patients negative for aPL (n = 41), we verified a high homogeneity in the treatments administered, so that almost all the patients received A/A, HCQ, and prednisone. We could only distinguish a number of patients treated with immunosuppressive drugs (n = 10) from those not taking that drug (n = 31) (Supplemental Table VI). Comparative analyses between those groups demonstrated a significant increase in the expression of miR146 and miR155 in monocytes of SLE patients that received immunosuppressive therapy (Azathioprine or Mycophenolate mofetil) in relation to the ones non-treated with those drugs (data not shown).

### Biomarkers of miRNA biogenesis are underexpressed in neutrophils from APS and SLE

**Figure 3:**
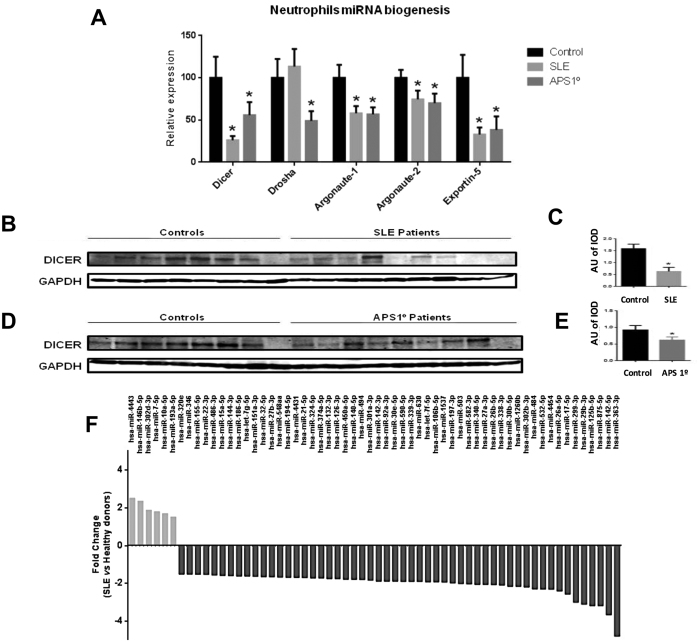
Biomarkers of miRNA-biogenesis in neutrophils from APS and SLE patients and comparison of the miRNA expression profiles in neutrophils from SLE patients’ vs healthy donors. (**A**) Relative mRNA expression levels of miRNA biogenesis proteins, including *Dicer*, Drosha, Ago1, Ago2 and Exportin-5, evaluated in isolated neutrophils from all the subjects included in the study (23 APS, 64 SLE, and 56 healthy donors) by qRT-PCR. (**B**) Representative Western blots of protein Dicer detection in neutrophils from 9 SLE patients and 8 healthy donors performed in triplicate as described in the Materials and methods section. (**C**) The bar graphs show mean protein expression levels ± SEM –expressed as arbitrary units (AU) of integrated optical density (IOD)- from all the SLE patients and controls included in the study. (**D**) Representative Western blots of protein Dicer detection in neutrophils from 9 primary APS patients and 8 healthy donors. (**E**) The bar graphs show mean protein expression levels ± SEM from all the primary APS patients and the controls included in the study. Asterisks (*) indicate significant differences (at P < 0.05) *vs* healthy donors. (**F**) NanoString assays were performed as described in Materials and Methods. miRNAs showing a fold change of at least 1.5 between patients and healthy donors are represented.

The mRNA expression levels of miRNA biogenesis proteins (Dicer, drosha, Ago-1, Ago-2 and Exportin 5) were found significantly reduced (except for drosha in SLE) in neutrophils from APS and SLE patients (Fig. 3A). No changes were found in monocytes (data not shown). Analysis of Dicer expression by Western blot showed that the expression of this protein appeared reduced by 68% and 34% in neutrophils from SLE and APS patients, respectively (Fig. 3B–E), indicating a potential defect in miRNA biogenesis pathway in neutrophils of these patients.

### NanoString nCounter assay showed that most miRNAs are significantly underexpressed in SLE neutrophils

The nCounter profiling identified the expression of 163 miRNAs in neutrophils from SLE patients and healthy donors. Of these, 129 (79,1%) were underexpressed and 34 (20,9%) were overexpressed in neutrophils from SLE patients. Among them, 60 miRNAs showed at least 1, 5-fold change between patients and healthy donors (Fig. 3F). Those results suggest that the alteration in the expression of miRNA biogenesis proteins might lead to reduced miRNAs expression in neutrophils from SLE patients.

### Non-autoimmune patients with previous thrombotic events show differential miRNAs alteration than APS and SLE patients

In order to assess whether the altered expression of the miRNA evaluated was linked to the thrombophilic state of APS and SLE patients, or was a sign of the immune activation, an additional control group, including 20 patients with thrombosis but without aPL was evaluated.

The expression levels of the selected miRNAs in neutrophils were not altered in relation to healthy donors, except for miR125b, which appeared significantly increased (Supplemental Fig. 3A). In monocytes, miR125a, miR-222, miR-125b, miR146a, and miR155 were found significantly reduced (Supplemental Fig. 3B).

### Positivity for aPL in SLE patients influences the expression of miRNAs associated to thrombosis development

**Figure 4:**
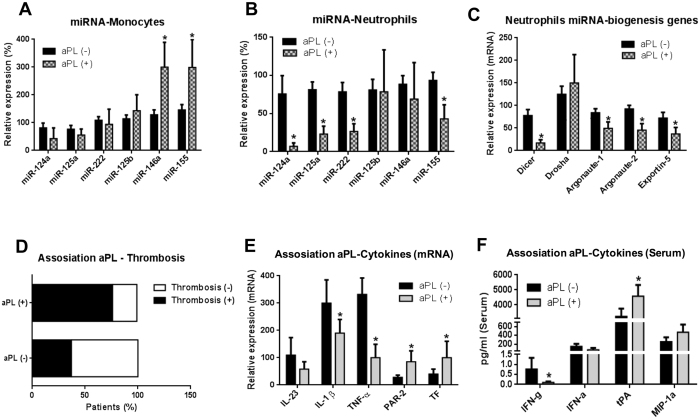
Expression of miRNAs, miRNA-biogenesis proteins, and inflammatory mediators in SLE patients positive for aPL versus SLE patients negative for aPL. SLE patients were sub-grouped according to aPL positivity, and statistical analyses were performed to identify the changes occurred in the selected miRNAs (Student’s test). miRNA levels were measured in monocytes (**A**) and neutrophils (**B**) isolated from SLE patients with or without aPL by qRT-PCR, and normalized with U6 snRNA. Statistical significance was taken as p < 0.05. (**C**) Relative mRNA expression levels of miRNA biogenesis proteins, including Dicer, Drosha, Ago1, Ago2 and Exportin-5. (**D**) Relationship between the positivity for aPL and occurrence of thrombotic events in SLE patients. (**E**) Relationship between the positivity for aPL and the gene expression of inflammatory parameters in monocytes from SLE patients. (**F**) Relationship between the positivity for aPL and the expression of inflammatory parameters in the plasma from SLE patients.

SLE patients were sub-grouped according to aPL positivity, and statistical analyses were performed to identify the changes occurred in the selected miRNAs –linked to CVD- evaluated in this study. In monocytes, a significant increase in two miRNAs (miR-146a and miR-155) was demonstrated in aPL-positive SLE in relation to the aPL-negative SLE patients (Fig. 4A). aPL-(+) SLE patients showed a significant reduction in the expression of four out of the six miRNAs in neutrophils in relation to aPL-(−) SLE patients (miR-124a, miR-125a, miR-222, and miR155) (Fig. 4B). Accordingly, a number of miRNA-biogenesis genes were also found reduced in those patients (Fig. 4C).

In addition, aPL-positivity in SLE patients was linked to the development of thrombosis (around a 70% of SLE patients showing high titres of aPL had suffered at least one thrombotic episode), while a reduced number of aPL-(−) SLE patients had suffered thrombotic events (Fig. 4D) (see Supplemental Tables V and VI for further details). In accordance with that results, proinflammatory profile also differed among aPL(−) and aPL(+)-SLE patients, so that while in aPL(−)-SLE patients cytokines such as IFNγ, IL-23, IL-1ß and TNFα were more prevalent, in aPL-(+) SLE patients an increased expression of tPA, PAR-2 and TF was noticed (Fig. 4E,F).

We further analysed differences in miRNAs expression in SLE patients that had suffered previous thrombotic events and those without thrombosis in relation to healthy donors (Supplemental Fig. 5) SLE patients T(+) showed significantly reduced levels of all the miRNAs evaluated in neutrophils in comparison with healthy donors, as well as reduced expression of miR124a and miR125a, and increased expression of miR-155 in monocytes. Proteins related to miRNA biogenesis were found also significantly reduced in neutrophils.

In SLE patients T(−) we found significantly reduced levels of three of the six miRNAs evaluated in neutrophils, while in monocytes we identified reduced expression of miR124a and miR125a, as well as elevated expression of miR146a and miR155. Proteins related to miRNA biogenesis were, like in SLE patients T(+), significantly reduced in neutrophils.

### Prothrombotic, Inflammatory, and oxidative stress parameters are deregulated in APS and SLE patients

As reported in previous studies^3^,^6^,^16^, in our set of patients, APS monocytes showed increased cell surface expression of TF and PAR2 (Supplementary Table SI). APS patients also displayed increased plasma levels of IL-8, MCP-1, and tPA. SLE patients demonstrated increased levels of monocyte TF and PAR2, as well as augmented plasma levels of IL-6, IL-8, IL-17, IL-23, MCP-1, and t-PA.

Peroxide/peroxinitrites production was notably increased in monocytes and neutrophils of APS and SLE patients. In line with this, the percentage of cells with altered ΔΨm was found significantly increased in monocytes and neutrophils. A prominent increase in the activity of monocyte mitochondrial SOD was found in APS and SLE patients compared with healthy donors. Yet, the activities of CAT and GPx were notably reduced in both monocytes and neutrophils from APS patients, as well as in neutrophils from SLE patients.

### Correlation and association studies

Correlation and association studies in SLE patients showed that the expression levels of the analyzed miRNAs in neutrophils were linked with some parameters related to autoimmunity, such as the presence of elevated autoantibody titers (i.e. anti-dsDNA over 50 and ACA-IgG over 25 GPL), and with plasma and cellular levels of different proinflammatory and proatherothrombotic proteins (Supplementary Table SII). Correlation analyses with markers related to oxidative stress further showed significant negative correlations with peroxide levels and with the percentage of cells with altered ΔΨm, as well as positive correlations with CAT activity in neutrophils of SLE patients. Some of these correlations were also found among various miRNAs in monocytes of SLE patients.

Association studies showed that the occurrence of a thrombotic event was related to low expression levels of 4 of the 6 evaluated miRNAs in neutrophils as well as to both, the reduced levels of miR-125a and the increased expression of miR-155, in monocytes from SLE patients (Supplementary Fig. 1A). The reduced expression of the 6 selected miRNAs in neutrophils and the altered expression of miR-146a and miR-155 in monocytes were also found associated with a pathological increase in the CIMT in these patients (Supplementary Fig. 1B).

As in the case of SLE patients, correlation studies in APS patients showed that the expression levels of some miRNAs differentially expressed in neutrophils and monocytes, significantly correlated with parameters related to autoimmunity, as well as with different proteins related to thrombosis, inflammation, and oxidative stress (Supplementary Table SIII).

We also found in APS patients an association between the occurrence of thrombotic events and the altered levels of a number of miRNAs in neutrophils and monocytes (Supplementary Fig. 2A). Levels of some of the miRNAs differentially expressed in monocytes and neutrophils from APS patients were further associated with a pathological increase in the CIMT (Supplementary Fig. 2B).

Furthermore, association studies showed that the occurrence of thrombotic events and a pathological increase of CIMT were also associated with low levels of proteins related to miRNA biogenesis in both SLE and APS patients (Supplementary Figs 1C,D and 2C,D, respectively).

### Anti-phospholipid-IgG antibodies and anti-dsDNA-IgG modulate both the expression of selected miRNAs and the expression of Dicer

**Figure 5:**
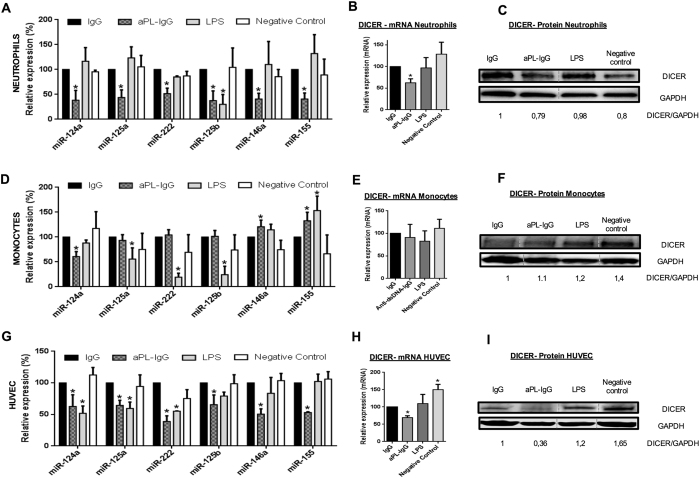
*In vitro* effects of aPL-IgG antibodies on both, the expression of selected microRNAs, and the expression of Dicer. Monocytes and neutrophils isolated from healthy donors, as well as primary human umbilical vein endothelial cells (ECs) were treated *in vitro* with anti-phospholipid IgG isotype antibodies (aPL-IgG) purified from APS patients’ serum, or with LPS as positive control, or cell culture medium as negative control, or LPS as positive control, or culture medium as negative control, or synthetic human IgG. (**A,D,G**) Relative expression levels of selected miRNAs in neutrophils, monocytes, and ECs, respectively. Values are the means and SEM of 4 independent experiments. Significant differences (*P < 0.05) *vs* neutrophils or monocytes or ECs treated with synthetic IgG. (**B,E,H**) Relative expression levels of *Dicer* mRNA in neutrophils, monocytes, and ECs treated with aPL-IgG, LPS, culture medium, or with synthetic IgG of 4 independent experiments. Significant differences (*P < 0.05) *vs* cells treated with synthetic IgG. (**C,F,I**) Representative Western blotting results of 4 independent experiments showing Dicer expression after the treatment indicated in neutrophils, monocytes, and ECs. Lanes were run on the same gel under the same experimental conditions but were non-contiguous. Cropping lines are used in the figure. Full-length blots are presented in Supplementary Fig. S6A.

**Figure 6:**
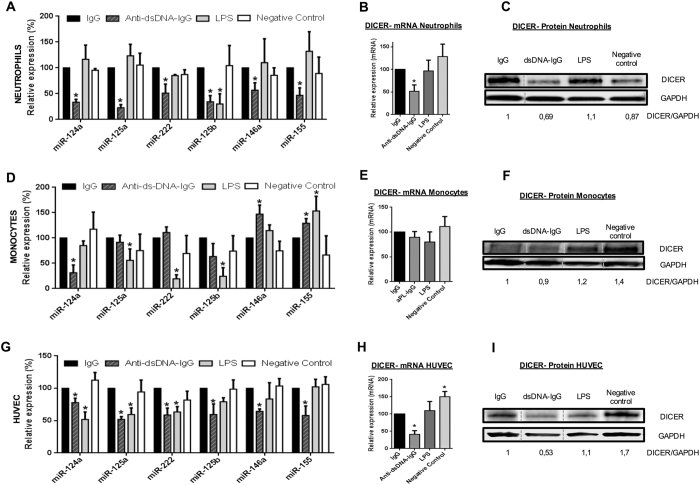
*In vitro* effects of anti-dsDNA-IgG antibodies on both, the expression of selected microRNAs, and the expression of Dicer. Monocytes and neutrophils isolated from healthy donors, as well as primary human umbilical vein endothelial cells (ECs) were treated *in vitro* with anti-dsDNA-IgG antibodies purified from SLE patients’ serum, or LPS as positive control, or culture medium as negative control, or with synthetic human IgG. (**A,D,G**) Relative expression levels of selected miRNAs in neutrophils, monocytes, and ECs, respectively. Values are the means and SEM of 4 independent experiments. Significant differences (*P < 0.05) *vs* neutrophils or monocytes or ECs treated with synthetic IgG. (**B,E,H**) Relative expression levels of *Dicer* mRNA in neutrophils, monocytes, and ECs treated with anti-ds-DNA-IgG, LPS, culture medium, or with synthetic IgG of 4 independent experiments. Significant differences (*P < 0.05) *vs* cells treated with synthetic IgG. (**C,F,I**) Representative Western blotting results of 4 independent experiments showing Dicer expression after the treatment indicated in neutrophils, monocytes, and ECs. Lanes were run on the same gel under the same experimental conditions but were non-contiguous. Cropping lines are used in the figure. Full-length blots are presented in Supplementary Fig. S6A.

The expression of all miRNAs analysed was significantly reduced in neutrophils treated with either aPL-IgG or anti-dsDNA-IgG compared to those treated with synthetic human IgG (Figs 5A and 6A). Both autoantibodies also caused a significant decrease in the expression of Dicer in this cell type (Figs 5B,C and 6B,C). In monocytes, treatment with aPL-IgG or anti-dsDNA-IgG promoted a significant reduction in the levels of miR-124a, as well as increased expression of miR-146a and miR-155 (Figs 5D and 6D). Levels of Dicer remained unchanged (Figs 5E,F and 6E,F). Likewise, in ECs both IgG-aPL and anti-dsDNA antibodies promoted a significant inhibition in the expression of microRNAs evaluated (Figs 5G and 6G), as well as a reduced expression of the biogenesis protein DICER at both, mRNA and protein levels (Figs 5H,I and 6H,I). A trend to a reduction in other biogenesis proteins was further observed (Supplemental Fig. 4A). Accordingly, both autoantibodies induced upregulation of MCP-1, TF, and VCAM-1, and downregulation of eNOS, relevant markers of endothelial dysfunction, and potential targets of the miRNAs evaluated (Supplemental Fig. 4B).

The administration of a positive control for inflammatory pathways and immune activation, such as LPS^27^, further promoted significant changes in the miRNAs and biogenesis proteins evaluated, although that changes were occasionally divergent from those induced by both autoantibodies, thus suggesting a differential modulation of cellular activation.

### Transfection with miR-124a and/or miR-125a mimics promoted decreased expression of inflammatory markers and their intracellular pathways

**Figure 7:**
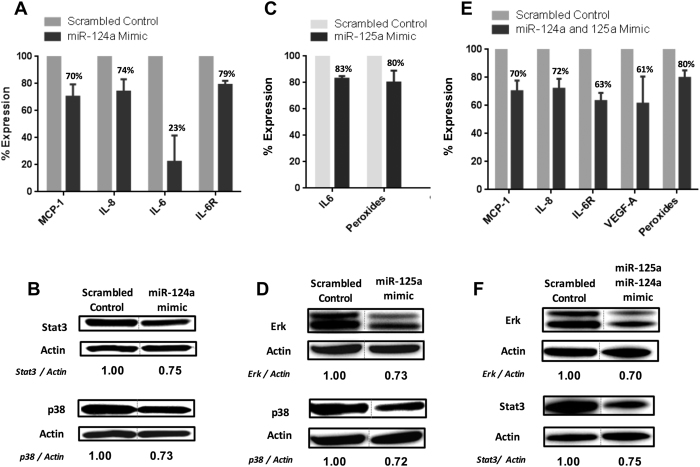
Transfection with miR-124a and/or -125a mimics promoted decreased expression of inflammatory markers and their intracellular pathways. Monocytes isolated from healthy donors were transfected with 100 nmol/L miR-124a, miR-125a mimics -either separately or in conjunction-, and a non-specific control (scrambled) by using siPORT^TM^ NeoFX^TM^ transfection agent following manufacturer’s protocols. *Forty*-*two* hours after transfection cells were activated with 10 mg/mL LPS for 6 h, and potential targets were analyzed by qRT-PCR (*MCP-1, IL-8, IL-6, IL-6R and VEGF*), Flow cytometry (peroxides), and Western blot (STAT3, ERK, and p38MAPK). Data, obtained from 4 independent transfection experiments, were expressed as changes relative to the values of the cells transfected with scrambled control, and set as 100%. (**A,B**) Changes promoted by transfection of monocytes with miR-124a mimic. (**C,D**) Changes promoted by transfection of monocytes with miRNA mimic for miR-125a. (**E,F**) Changes promoted by simultaneous transfection of monocytes with miR-124a and miR-125a mimics. Western blots show representative results from 4 separated experiments with similar results. Lanes were run on the same gel under the same experimental conditions but were non-contiguous. Cropping lines are used in the figure. Full-length blots are presented in Supplementary Fig. S6B for key data.

Monocyte transfections with miR-124a and miR-125a mimics, either separately or simultaneously, caused a reduction in the expression of target molecules related to the atherothrombotic process in APS and SLE, such as MCP-1, IL-6, IL6R, IL-8, ERK, STAT-3, p38 MAPK and peroxides (Fig. 7A–F). The simultaneous transfection with both pre-miRNAs did not potentiate the inhibitory effect caused by each miRNA mimic administered separately, but increased the global number of molecules targeted.

## Discussion

There is increasing evidence connecting an imbalance between various proinflammatory mediators with higher CVD risk in APS and SLE patients^28^,^29^. Accordingly, in previous reports, we demonstrated that prothrombotic and inflammatory molecules, oxidative stress markers, and mitochondrial dysfunction, were associated with premature atherosclerosis and/or the occurrence of thrombotic events in SLE^9^,^16^. Moreover, the same inflammatory/oxidative molecules seemed to orchestrate the atherothrombotic status present in APS patients^3^, suggesting specificity of the CV comorbidity in these autoimmune patients. These preliminary data encouraged us to come across a new search of mechanisms orchestrating those processes, such as the study of miRNAs that specifically modulate those targets, and to analyze their expression and modulation in the setting of APS and SLE.

The present study demonstrated, for the first time, the altered expression of a number of miRNAs directly involved in atherothrombosis, and their modulation by effect of specific autoantibodies in both pathologies.

Previous studies by our group supported the occurrence of a specific gene profile in SLE patients positive for aPL in relation to those negative for aPL, which was further associated to atherothrombosis^16^. Moreover, that study linked the presence of a high CVD risk in the formers with the overexpression of various prothrombotic and proinflammatory mediators (i.e. PAR-2, TF, MCP-1, or tPA). However, the classical inflammatory cytokines (ie, IFNγ, TNFα, IL-1β, IL-6) that orchestrate common pathophysiological processes in SLE (ie, nephritis, skin manifestations, neurological affectations, etc) were more specifically linked to aPL-(−) SLE patients, suggesting molecular and cellular specificity of the CV comorbidity. The present study further confirmed this hypothesis, showing that aPL-(+) SLE patients displayed an specific dysregulation of a number of miRNAs in relation to aPL-(−) SLE patients. In accordance with that results, proinflammatory profile also differed among aPL(−) and aPL(+)-SLE patients in the same way as described above, including a number of targets of the measured miRNAs. That data supported the direct linkage among the altered expression of specific microRNAs, the presence of aPL, and the increased risk of atherothrombosis in this group of SLE patients.

The analysis of miRNA expression in SLE patients that had suffered previous thrombotic events and those without thrombosis in relation to healthy donors, demonstrated an altered expression of miRNAs independent of the thrombophilic state. In addition, those changes were directly related to the positivity for both aPL and anti-dsDNA antibodies, as indicated by association studies. Furthermore, their expression was modulated by *in vitro* stimulation with both autoantibodies. Thus, those miRNAs could be considered mostly markers of immune activation in lupus, which in turn might lead to a prothrombotic and proinflammatory status in SLE patients.

In line with those results, the analysis of microRNAs in patients with previous thrombotic events but without an autoimmune-related profile revealed a differential pattern of expression, with almost no changes in neutrophils, and a significant reduction in the majority of microRNAs in monocytes in relation to healthy donors. Previous studies have reported a substantial involvement of the evaluated microRNAs on CVD^30^,^31^,^32^,^33^,^34^. Yet, thrombotic patients displayed a specific profile of miRNA expression that would reflect a differential and specific mode of regulation and activity in relation to autoimmune patients, on which autoantibodies most probably play a key role.

Among the miRNAs selected for characterization in APS and SLE patients, miR-146a and miR-155 have been previously shown to play critical roles in lymphocyte development, differentiation, function, and also in the control of both innate and adaptive immune responses. Deregulated miR146a and miR-155 expression and/or function have been associated with various autoimmune diseases including SLE and rheumatoid arthritis (RA)^18^,^35^. In our hands, miR-146a and miR-155 were found downregulated in neutrophils, while their expression was found significantly increased in monocytes from both SLE and APS patients, and a trend to a reduction was observed in lymphocytes. Moreover, their overexpression correlated with levels of ACA-IgG and anti-dsDNA antibodies, as well as with increased plasma levels of pro-inflammatory molecules and oxidative stress markers. *Our in vitro* studies further confirmed the *in vivo* correlations, demonstrating that both aPL-IgG and anti-dsDNA antibodies promoted a significant reduction of these miRNAs in neutrophils and a prominent increase in monocytes.

Contrary to our results, a previous study reported that miR-146a was underexpressed in PBMCs of SLE patients, and negatively correlated with type I interferon and clinical disease activity^36^. That apparently contradictory data may be related to a distinct miRNA expression in different leucocyte subsets, which might differentially contribute to the overall inflammatory status in both autoimmune diseases. Indeed, a recent study has shown that monocytes overexpressing miR-146a display a dampened inflammatory response^37^ suggesting that this miRNA might play a role as a molecular brake on inflammation in the setting of APS and SLE. MiR-155 levels have been reported to be elevated in B but low in T cells from SLE patients^38^ and deleting miR-155 prevents the production of harmful antibodies, alleviating lupus-like disease in mice^39^. In our study, as in the case of miR-146a, the over-expression of miR-155 found in APS and SLE monocytes might act as a protective factor against the inflammatory effect of autoantibodies, as previously reported in the setting of RA^40^,^41^.

On the contrary, significant reduction was found in miR-125a levels in both neutrophils and monocytes from APS and SLE patients. This miRNA is involved in the inflammatory chemokine pathway of SLE, and increases the expression of inflammatory chemokine RANTES by targeting KLF13 in SLE^42^. In the present study, reduced miR-125a levels correlated with oxidative stress markers, inflammatory and prothrombotic molecules, and autoimmunity parameters, and were also related to the occurrence of thrombotic events in both APS and SLE patients. Furthermore, miR-125a overexpression in monocytes significantly reduced peroxides levels, IL-6 expression, and intracellular inflammatory signaling kinases, including ERK and p38 MAPK.

It is known that miR-124a targets monocyte chemoattractant protein 1 (MCP-1)^43^, which is significantly elevated in APS, SLE, and RA patients, and involved in the CV pathogenesis of that autoimmune conditions^3^,^9^,^44^. In the present study, miR-124a was found significantly reduced in both neutrophils and monocytes from APS and SLE patients, and related to thrombotic events and a pathological CIMT. MiR-124a expression was strongly downregulated by autoantibodies from both autoimmune conditions, and its overexpression in monocytes downregulated not only MCP-1 but also IL-8, IL-6, IL-6R, STAT3 and p38 MAPK. Thus, miR-124a, together with miR-125a seems to orchestrate the inflammatory status that underlies the pathophysiology of CVD in APS and SLE. Furthermore, our overall data unveiled new roles and targets for both miRNAs in those autoimmune diseases.

Some miRNAs are emerging as being associated with or localized in mitochondria, could play a direct role in regulating mitochondrial function^45^,^46^,^47^, and their modulation may be involved in various pathological processes, including inflammation. The present study reports an altered expression of some of these miRNAs, in APS and SLE patients, that is associated to the mitochondrial dysfunction and the pro-oxidative status present in those diseases. Thus, the expression of miR-125a, miR-155 and miR-146a correlated with the levels of peroxides as well as with the increased percentage of cells with altered ΔΨm and the increased activity of mitochondrial SOD. Moreover, transfection of monocytes with miR-125a mimics promoted a reduction in peroxides production. Thus, the up-regulation of those miRNAs could mediate the loss of mitochondrial integrity and function, thus contributing to the pro-oxidative status of APS and SLE.

In general, APS and SLE patients are treated with anticoagulants/anti-agregants, antimalarial agents, corticosteroids, and immunosuppressive medications. All of them have been shown to influence miRNA expression, an epigenetic mechanism that might help to delineate the mechanisms underlying their effects. Thus, studies have demonstrated that HCQ altered the expression of miR-21 and miR-let-7a in PBMCs from NZB/W lupus mice, partially explaining its anti-inflammatory effects^48^. Similarly, in NZB/W mice treated with prednisone, expression levels of miR-lt-7a, miR 21, miR 146a and miR155 all changed^48^. Also immunosuppressants such as metothrexate have been confirmed to alter the epigenetic status in other diseases, such as cancer^49^. With that premises, in our cohort of patients, the putative effects of the administered drugs on the expression of the miRNAs evaluated in APS and SLE patients were assessed. As a general feature, we found a high homogeneity in the treatments administered, so that most APS patients were treated with anti-agregants/anticoagulants and antimalarials, while almost all the SLE patients received A/A, HCQ and prednisone. We could also identify a number of SLE patients further treated with immunosuppressive drugs. Comparative analyses demonstrated a significant increase in the expression of miR146 and miR155 in monocytes of SLE patients receiving immunosuppressive therapy in relation to the ones non-treated with those drugs. As stated above, monocytes in our cohort of SLE patients showed a significant overexpression of both miR146 and miR155. That overexpression was hypothesized to act as a protective factor against the inflammatory effects of autoantibodies. Due to the anti-inflammatory nature of immunosuppressive drugs, it might be speculated that the even increased expression of both miRNAs in immunosuppressant-treated patients could further contribute to repress inflammation.

A remarkable heterogeneity in autoantibody profile and clinical presentation is well known in SLE patients. The two prominent class of autoantibody populations in SLE are targeted against either dsDNA or RNA-associated proteins. However, the basis for this distinctive autoantibody profile and its regulation in SLE patients is poorly understood. In a previous study we demonstrated that the occurrence of thrombotic events in SLE was associated with factors related to autoimmunity, including titers of aCL-IgG and anti-dsDNA^9^. By using an *in vitro* approach, in the present study we have demonstrated that anti-dsDNA antibodies not only paralleled the altered appearance of some proinflammatory mediators in SLE, but also acted as direct modulators of the expression of a number of miRNAs that, in turn, orchestrated their expression. Our results were further supported by a recent study that reported a differential and varying miRNA expression profile in subsets of SLE patients characterized on the basis of distinct autoantibodies repertoires. Using the QIAGEN’s Ingenuity Pathway Analysis (IPA^®^, QIAGEN Redwood City, www.qiagen.com/ingenuity), they identified cell cycle- and cytoskeleton remodeling-related events as the main targets of miRNAs dysregulated in anti-ENA+ patients, whereas miRNAs dysregulated in anti-dsDNA+ patients were found to be involved in multiple cytokine signaling pathways. Our study further delineated that association^50^.

miRNAs expressed in the vasculature play important roles in CVD^51^. Vascular inflammation is an early step in atherogenesis, and many miRNAs are induced in inflamed ECs. Thus, previous studies reported that TNFα treatment decreased miR181b expression (promoting induction of adhesion molecules such as VCAM-1)^52^ and induce miR-17, miR31, miR155, miR-221 and miR222 expression (regulating the adhesion of neutrophils and T cells to activated ECs, as well as the proliferation and migration of ECs)^53^,^54^. The present study further showed that aPL-IgG and anti-ds-DNA-IgG autoantibodies downregulated in ECs all the miRNAs evaluated, also related to vascular dysfunction in the setting of APS and SLE, as demonstrated by the induced upregulation of MCP-1, TF, and VCAM-1, and the downregulation of eNOS in ECs. Thus, analyzed miRNAs might also play a pivotal role on endothelial function in both autoimmune conditions.

The generation of miRNAs is mainly dependent on the RNAse III enzyme Dicer, the levels of which vary in different normal cells and in disease states^55^. It has been shown that interruption of miRNA biogenesis machinery (through spontaneous or induced DICER-deficiency) contributes to the abnormal T and B cell development, as well as to altered endothelial function, leading to vascular dysfunction and systemic autoimmune diseases^56^. In this study, we further demonstrated a reduced expression of DICER, along with other proteins related to miRNA biogenesis, in neutrophils from APS and SLE patients. That reduced expression was associated with clinical aspects of these diseases, including the occurrence of thrombotic events and the presence of a pathologic CIMT. Moreover, the analysis of a wide set of miRNAs in neutrophils from SLE patients demonstrated the underexpression of approximately an 80% of them when compared with neutrophils from healthy donors, thus supporting the hypothesis that interrupted miRNA biogenesis plays a key role in SLE development and progression.

Previous studies demonstrated that Dicer protein expression can be inhibited by multiple types of stress, including reactive oxygen species, phorbol esters and the Ras oncogenes, as well as IFN type I^57^. Accordingly, the presence of a chronic inflammatory and oxidative status in APS and SLE patients might contribute to the reduced levels of DICER found in neutrophils. Our *in vitro* studies further demonstrated that DICER mRNA and protein expression levels, in both neutrophils and endothelial cells, were downregulated by effect of specific autoantibodies of APS and SLE, which are as well responsible for the altered expression of proinflammatory and oxidative stress markers. Those molecules in conjunction might thus influence DICER inhibition.

Transfection studies in monocytes with miR-124a and/or miR-125a showed a downregulation of a number of inflammatory markers associated with the pathophysiology of CVD in APS and SLE. Moreover, we noticed that the simultaneous transfection with both miRNAs did not potentiate the effect caused by each miRNA administered separately, but increased the number of molecules targeted. Of note, the inhibitory effect was moderate. It has been widely demonstrated that while a single miRNA may target hundreds of genes, the effect of miRNAs on individual target protein synthesis is mild and moderate^56^. However, given that a specific inflammatory-related-gene may contain targeting sites for different miRNAs, it is plausible that multiple SLE/APS-associated miRNAs, rather than a single miRNA, synergistically act together to alter the overall inflammatory status in these patients.

Taken together, the current study has revealed that: 1. Specific miRNAs might act as potential biomarkers of immune activation and atherothrombosis in APS and SLE patients. 2. miRNA biogenesis is significantly altered in neutrophils of APS and SLE patients and associated to both, the presence of a pathologic CIMT, and the occurrence of thrombotic events. 3. Anti-dsDNA and aPL antibodies regulate CVD in APS and SLE, at least partially, by modulating the biogenesis and the expression of microRNAs.

## Methods

### Patients

Eighty seven patients, 23 with primary APS, and 64 with SLE, as well as 56 healthy donors were included in the study (during a period of 48 months). All experimental protocols were approved by the ethics committee of the Reina Sofia Hospital in Cordoba and written informed consent was obtained. All methods were carried out in accordance with the approved guidelines. Subjects were selected among the patients with stable disease for more than six months, without infections, abortions, thrombosis, or changes in their treatment protocol. None of the healthy controls had a history of autoimmune disease, bleeding disorders, thrombosis, or pregnancy loss. The characteristics of patients and controls are shown in Table 1.

We further studied 20 patients with thrombosis but without aPL (14 non-pregnant women and 6 men, mean age 45 (range: 24–63 years), including patients with objectively verified thrombotic events: 8 deep venous thrombosis and 12 thrombosis in intra-cerebral vessels).

### B-Mode Ultrasound IMT Measurements

All patients and controls underwent B-mode ultrasound imaging for CIMT (carotid intimate media thickness) measurements as previously described^9^,^16^.

### Blood samples

The collection of peripheral venous blood samples for obtaining plasma, serum, and for purifying monocytes (non-monocytes depleting kit, Miltenyi Biotech, Bergisch Galdbach, Germany), lymphocytes, and neutrophils (dextran sedimentation) was performed as described elsewhere^9^,^16^.

### Flow cytometry analyses, and analysis of oxidative stress biomarkers in purified leukocytes and plasma

Flow cytometric analysis was performed in monocytes as previously described^9^ using a FACScan (BD Biosciences, San Jose, CA,). The BD Cytofix/Cytoperm fixation/permeabilization kit was used to analyse the intracellular expression of some cytokines (i.e. IL-6, IL-8, MCP-1), according to manufacturer’s instructions.

CD40L, IFNα, IFNγ, IL-1, IL-2, IL-6, IL-8, IL-10, IL-17, IL-23, MCP-1, MIP-1α, TNFα, tPA, VEGF-A, and sP-selectin levels were quantified in sera using a cytofluorimetry-based ELISA system (Flowcytomix, Bender Medsystem GmbH, Austria)^3^.

Oxidative stress biomarkers were analysed in purified leukocytes using a dual-laser FACSCalibur (Becton Dickinson, Mountain View, CA) as previously described^3^,^9^.

Mitochondrial SOD activity (Mn-SOD), Catalase (CAT) activity and Glutathione peroxidase (GPx) activity were assayed in cell lysates using specific kits (Cayman Chemical Company, ciudad, MI).

### Western Blotting

Dicer, p38 MAPK, Erk, STAT-3, GAPDH and actin protein levels were determined by Western blotting^3^,^9^, using specific antibodies (Abcam and Santa Cruz Biotechnology).

### *In silico* studies

Databases and algorithms of miRNA target prediction were used for the search of miRNAs targeting prothrombotic and proinflammatory mediators. We used TargetScan (release 5.1: http://www.targetscan.org), which provides the prediction results computed by the TargetScanS algorithm^58^, PicTar (http://pictar.mdc-berlin.de)^59^, and miRanda http://www.microrna.org/microrna/home.do)^60^.

### RNA isolation and qRT-PCR for mRNA and microRNA expression

Total RNA from lymphocytes, monocytes, and neutrophils was extracted using TRI Reagent (Sigma, St Louis, MO) following manufacturer’s recommendations. MiRNA biogenesis modulators Dicer, Drosha, Argonaute-1, Argonaute-2, Exportin-5, and other inflammatory molecules were quantified as previously described^9^,^16^.

For quantification of mature miRNA levels, trizol purified RNA was used. cDNA was synthesized from 200 ng RNA using individual miRNA-specific RT primers contained in the TaqMan^®^ MicroRNA Reverse Transcription Kit. Each cDNA was amplified using the TaqMan^®^ MicroRNA assays together with TaqMan^®^ Universal PCR Master Mix, No AmpErase^®^ UNG (Life Technologies, Madrid, Spain). The 2^−ΔCt^ method was used to calculate the relative abundance of miRNAs compared with U6 snRNA expression.

### NanoString nCounter assay

For miRNA expression data generation, the NanoString human v2 array, which contains 800 miRNA probes, was used. Pools with RNA purified of neutrophils from 5 SLE patients and from 5 healthy donors were performed. A total of 100 ng RNA input was used per sample and conditions were set according to the manufacturer’s recommended protocol (NanoString Technologies; Seattle, WA). Data were normalized by the geometric mean of all targets using the nSolver software.

### Purification of IgG and *in vitro* exposure of white blood cells and endothelial cells to aPL antibodies

IgG from the pooled sera of 7 patients with APS (characterized by high titres of anti-cardiolipin –aCL- and anti-ß2GPI antibodies) was purified by protein G-Sepharose high-affinity chromatography (MAbTrap kit; Amersham Biosciences) following the manufacturer’s recommendations. Briefly, MAbTrap Kit contains a HiTrap™ column prepacked with Protein G Sepharose™, a Type III Fc receptor that binds to the Fc region of all IgG fractions present in serum or plasma by a chromatographic method. Anti-ß2GPI and IgG-aCL activities of purified IgG were confirmed by enzyme-linked immunosorbent assays (QUANTA Lite^®^ ß2GPI-IgG and QUANTA Lite^®^ ACA IgG III kits, Inova Diagnostics; San Diego, CA, USA).

For *in vitro* studies, monocytes and neutrophils purified from healthy donors were incubated either with 10 μg/ml LPS, synthetic human IgG (40 μg/mL) (Jackson InmunoResearch Laboratories, Inc, Newmarket, Suffolk, UK) or purified APS patient-IgG (40 μg/mL) for 6 hours at 37 °C.

Primary human umbilical vein endothelial cells (HUVECs) were purchased from Lonza Group Ltd (Basel, Switzerland) and cultured in Endothelial Basal Medium (EBM, Lonza, Walkersville, MD USA) supplemented with 10% fetal bovine serum (FBS, Lonza), 0.1% human epidermal growth factor (hEGF, Lonza), 0.1% hydrocortisone (Lonza), 0.1% Gentamicin-Amphotericin-B (GA-1000, Lonza), 0.4% bovine brain extract (BBE, Lonza), and 1% Zell Shield (Minerva Biolabs, GmbH, Berlin, Germany) at 37 °C and 5% CO_2_. Confluent cell monolayers were treated for 6 hours at 37 °C with aPL-IgG and anti-dsDNA antibodies, or with LPS or synthetic human IgG, as described above. All the experiments were performed on passage 4.

### Purification of anti-dsDNA IgG from SLE patients and *in vitro* culture of white blood cells and endothelial cells

Anti-dsDNA IgG antibodies from the pooled sera of 7 SLE patients (characterised by high titres of anti-dsDNA) were purified using a commercial kit (Quanta Lite-INOVA Diagnostics, San Diego, CA) through the ELISA-elution assay method (Referencia ATVB). Monocytes, neutrophils, and ECs, were then incubated with 10 μg/ml LPS, synthetic human IgG (40 μg/ml) or purified Anti-dsDNA IgG from the pooled sera of 5 SLE patients (40 μg/ml) for 6 h at 37 °C.

### Cell transfection

Monocytes purified from healthy donors were plated 24 hours before transfection in 6-well plates with complete medium without antibiotics (Opti-MEM, Life Technologies, Madrid, Spain). Cells were transfected with 100 nmol/L miRNA mimic (Life Technologies, Madrid, Spain) for miR-124a, miR-125a -either separately or in conjunction-, and a non-specific control (scrambled) by using siPORT^TM^ NeoFX^TM^ transfection agent (Life Technologies, Madrid, Spain). After 48 hours, cells were activated with 10 μg/mL LPS (Sigma-Aldrich, Madrid, Spain) for 6 h, and potential targets were analyzed. Data were expressed as changes relative to the values of the cells transfected with scrambled control.

### Statistical analysis

All data were expressed as mean ± SD. Statistical analyses were performed with SSPS 17.0 (SPSS Inc., Chicago, IL, USA). Following normality and equality of variance tests, comparisons were made by paired Student’s t test or alternatively by a non-parametric test (Mann–Whitney rank sum test). Correlations were assessed by Spearman rank correlation test and association studies were performed through Chi-square test. Differences were considered significant at *P* < 0.05.

## Additional Information

**How to cite this article**: Pérez-Sánchez, C. *et al*. ‘Atherothrombosis-associated microRNAs in Antiphospholipid syndrome and Systemic Lupus Erythematosus patients’. *Sci. Rep*. **6**, 31375; doi: 10.1038/srep31375 (2016).

## Supplementary Material

Supplementary Information

## Figures and Tables

**Table 1:** Clinical and Laboratory parameters of the Antiphospholipid Syndrome, Systemic Lupus Erythemat losus patients and the Controls.

	Healthy donors (N = 56)	APS patients (N = 23)	P^#^	SLE patients (N = 64)	*P*^*#*^
CLINICAL PARAMETERS*					
Females/Males	36/20	16/7		56/8	
Age, (years)	36.44 ± 9.19	44.90 ± 15		41.61 ± 12.21	n.s.
Anti-dsDNA positivity (%)	0	0	n.s.	18 (28%)	0.001
aCL IgG positivity (%)	0	5 (22%)	—	9 (14%)	
aCL IgM positivity (%)	0	3 (13%)		6 (9%)	
Anti-ß2GPI positivity (%)	0	9 (39%)		5 (8%)	
aCL IgG (GPL)	2.86 ± 4.32	21.52 ± 32.56		9.45 ± 17.99	0.015
aCL IgM (MPL)	6.05 ± 6.13	8.83 ± 10.72	0.023	18.24 ± 55.62	n.s.
Anti-ß2GPI (SGU)	4.07 ± 7.17	45.84 ± 94.37	n.s.	3.95 ± 8.26	n.s.
LA positivity (%)	0	7/10 (70%)	0.007	12/24 (50%)	
SLEDAI	0	—		1.69 ± 2.53	0.001
Thrombosis (%)	0	19 (83%)		26 (40%)	
Arterial Thrombosis (%)	0	16 (84%)		21 (81%)	
Venous Thrombosis (%)	0	5 (26%)		9 (35%)	
Fetal loss (%)	0	8 (35%)		7 (11%)	
Obesity (%)	1 (2%)	6 (26%)		15 (23%)	
Hypertension (%)	0	4 (17%)		10 (15%)	
Diabetes (%)	0	0		3 (5%)	
Smoking (%)	7 (13%)	4 (17%)		22 (34%)	
Hyperlipidemia (%)	3 (5%)	3 (13%)		10 (15%)	
Nephropathy (%)	0	0		17 (26%)	
Increased CIMT (%)	2 (4%)	9 (39%)		13 (20%)	
*Corticosteroids (%)*	0	2 (9%)		46 (71%)	
*Antimalarials (%)*	0	5 (22%)		43 (66%)	
*Anticoagulants/antiplatelets (%)*	0	18 (78%)		44 (68%)	
LABORATORY PARAMETERS*					
Total cholesterol (mg/dL)	191.18 ± 33.99	192.73 ± 39.37	n.s.	185.83 ± 33.60	n.s.
Cholesterol HDL (mg/dL)	55.74 ± 13.15	44.86 ± 10.83	0.001	51.78 ± 14.17	n.s.
Cholesterol LDL (mg/dL)	117.70 ± 29.01	122.57 ± 35.78	n.s.	113.95 ± 27.41	n.s.
Triglycerides (mg/dL)	86.89 ± 48.36	143.59 ± 138.95	n.s.	102.72 ± 44.47	n.s.
C reactive protein (mg/dL)	1.95 ± 5.40	3.40 ± 4.73	n.s.	3.06 ± 3.61	n.s.
Apolipoprotein A (g/L)	146.69 ± 28.24	135.55 ± 17.16	n.s.	146.87 ± 30.81	n.s.
Apolipoprotein B (g/L)	77.26 ± 16.69	84.70 ± 24.25	n.s.	77.52 ± 19.15	n.s
C3 (mg/dL)	124.27 ± 44.59	123.46 ± 29.19	n.s.	103.89 ± 28.22	0.010
C4 (mg/dL)	24.01 ± 9.23	21.74 ± 6.62	n.s.	17.65 ± 7.47	0.001

GPL indicates IgG phospholipid units; MPL, IgM phospholipid units; and SGU, standard IgG units. ^*^Except otherwise indicated, values are number of subjects and mean ± SD.
