# Supplementary material for: ‘Atherothrombosis-associated microRNAs in Antiphospholipid syndrome and Systemic Lupus Erythematosus patients’
Source: Sci Rep. 2016 Aug 9;6:31375. doi: 10.1038/srep31375 (PMC4977549; doi:10.1038/srep31375)
Supplement: Supplementary Information [file srep31375-s1.pdf]

'Atherothrombosis-associated microRNAs in Antiphospholipid syndrome and Systemic Lupus Erythematosus patients'

C. Pérez-Sánchez[1], MA Aguirre[1], P. Ruiz-Limón[1], N. Barbarroja[1], Y. Jiménez-Gómez[1], I. Arias de la Rosa[1], A. Rodriguez-Ariza[1], E. Collantes-Estévez[1], P Segui[1], F. Velasco[1], MJ Cuadrado[2], R. Teruel[3], R. González-Conejero[3], C. Martínez[3] and Ch. López-Pedrera[1*].

[1]Maimonides Institute for Research in Biomedicine of Cordoba (IMIBIC) / Reina Sofia University Hospital/University of Cordoba, Cordoba, Spain. [2]Lupus Research Unit, St Thomas Hospital, London, United Kingdom, [3]Regional Centre for Blood Donation, University of Murcia, IMIB-Arrixaca, Spain.

Supplementary Information

Flow cytometry analyses, and analysis of oxidative stress biomarkers in purified leukocytes and plasma, and western blotting.

Flow cytometry analysis

Flow cytometric analysis was performed in monocytes as previously described [6], using a FACScan (BD Biosciences, San Jose, CA,) and monoclonal anti-human FITC-conjugated TF antibodies (clone TF9-6B4, American Diagnostica, Greenwich, CT,), and anti-human FITC-conjugated PAR2 antibodies (Santa Cruz Biotechnology, Santa Cruz, CA). The BD Cytofix/Cytoperm fixation/permeabilization kit was used to analyse the intracellular expression of some cytokines (i.e. IL-6, IL-8, MCP-1), according to manufacturer's instructions.

FlowCytomix

CD40L, IFNα, IFNγ, IL-1, IL-2, IL-6, IL-8, IL-10, IL-17, IL-23, MCP-1, MIP-1α, TNFα, tPA, VEGF-A, and sP-selectin levels were quantified in sera using a cytofluorimetry-based ELISA system (Flowcytomix, Bender Medsystem GmbH, Austria). Two-colour cytometric analysis was performed using FACScalibur cytometer (Becton Dickinson Immunocytometry Systems (BDIS); San José, CA). Data were obtained and analysed using the FlowCytomix Pro software.

Determination of oxidative stress biomarkers in purified leukocyte subsets

Oxidative stress biomarkers were analysed in purified leukocytes using a dual-laser FACSCalibur (Becton Dickinson, Mountain View, CA). Test standardization and data acquisition analysis were performed using the CELL Quest software (Becton Dickinson, Mountain View, CA). A forward and side scatter gate was used for the selection and analysis of the different cell subpopulations.

For the assessment of ROS generation, including the joint detection of peroxides and peroxynitrites, cells were incubated with 5 µM DHR-123 at 37°C for 30 min in the dark. The JC-1 Mitoscreen assay (Becton Dickinson, Mountain View, CA) was used (final concentration 2 µM) to assess the mitochondrial membrane potential ($\Delta\psi_m$) according to manufacturer's instructions.

Determination of cell oxidative stress biomarkers
Mitochondrial SOD activity (Mn-SOD), Catalase (CAT) activity and Glutathione peroxidase (GPx) activity were assayed in cell lysates using specific kits (Cayman Chemical Company, ciudad, MI) according to manufacturer's instructions.

Western blotting
Dicer, p38 MAPK, Erk, STAT-3, GAPDH and actin protein levels were determined by Western blotting[6], using specific antibodies (Abcam and Santa Cruz Biotechnology).

RNA isolation and qRT-PCR for mRNA and microRNA expression

Total RNA from lymphocytes, monocytes, and neutrophils was extracted using TRI Reagent (Sigma, St Louis, MO) following the manufacturer's recommendations. The integrity of RNA was verified by optical density (OD) absorption ratio OD260/OD280 between 1.7 and 1.8.
For first strand cDNA synthesis, 1µg of total RNA was reverse transcribed using random hexamers (Roche Diagnostic, Indianapolis, IN) as primers and Transcriptor Reverse Transcriptase (Roche Diagnostic, Indianapolis, IN). Gene expression was assessed by real time PCR using a LightCycler Thermal Cycler System (Roche Diagnostics, Indianapolis, IN,). The reaction was performed, following the manufacturers protocol, in a final volume of 25µl.
The reactions consisted of an initial denaturing of 10 min at 95 ºC, then 40 cycles of 15 seconds denaturing phase at 95ºC, and 1 minute annealing and extension phase at 60ºC. A threshold cycle (Ct value) was obtained for each amplification curve and a ΔCt value was first calculated by subtracting the Ct value for human glyceraldehyde-3-phosphate dehydrogenase cDNA from the Ct value for each sample and transcript. Fold changes compared with the endogenous control were then determined by calculating 2-ΔCt. Every sample was performed in triplicate and negative controls were included in all the reactions. Test reproducibility for all investigated transcripts was less than 0.5% in inter-test experiments and even lower in intra-test experiments. MiRNA biogenesis modulators Dicer, Drosha, Argonaute-1, Argonaute-2, Exportin-5, as well as a number of inflammatory molecules were quantified.
For quantification of mature miRNA levels in lymphocytes, neutrophils and monocytes trizol purified RNA was used. cDNA was synthesized from 200 ng RNA using individual miRNA-specific RT primers contained in the TaqMan® MicroRNA Reverse Transcription Kit. Each cDNA was amplified using the TaqMan® MicroRNA assays together with TaqMan® Universal PCR Master Mix, No AmpErase® UNG (Life Technologies, Madrid, Spain). The $2^{-\Delta Ct}$ method was used to calculate the relative abundance of miRNAs compared with U6 snRNA expression and set as 100%.

NanoString sample preparation and data analysis.

A pool with RNA purified of neutrophils from 5 SLE patients and other pool with RNA purified of neutrophils from 5 healthy donors was performed. A total of 100 ng RNA input was used per sample and conditions were set according to the manufacturer's recommended protocol (NanoString Technologies; Seattle, WA). Briefly, mature miRNAs were ligated to a species-specific tag sequence (miRtag) via a thermally

controlled splinted ligation. The unligated miRtags were removed with enzymatic purification, and miRtagged mature miRNAs were then hybridized with an nCounter Human (V2) miRNA Expression Assay CodeSet overnight at 65°C. The unhybridized CodeSet was removed with automated purification performed on an nCounter Prep Station, and the remaining target:probe complexes were transferred and bound to an imaging surface. Counts of the reporter probes were tabulated for each sample by the nCounter Digital Analyzer, and raw data output was imported into nSolver (http://www.nanostring.com/products/nSolver). The nCounter® program uses a normalization factor to account for technical noise (such as variations in hybridization, purification, binding efficiency). Data were normalized by the genometric mean of all targets using the nSolver software. In addition, six internal negative controls and six positive controls were included in the nCounter miRNA Expression assays, to correct for background noise. The mean of the negative controls served as the medium stringency threshold for miRNA detection and high stringency was calculated by adding 2SD to the mean of the negative controls (threshold= mean+ 2SD).

Purification of anti-dsDNA IgG from SLE patients

Anti-dsDNA IgG antibodies from the pooled sera of 7 SLE patients (characterised by high titres of anti-dsDNA) were purified using a commercial kit (Quanta Lite-INOVA Diagnostics, San Diego, CA). Briefly, 100 μl of pooled sera were added to the microtitres plates coated with highly purified calf thymus ds DNA which allowed the binding of the antibodies (anti-ds-DNA). Next, anti-ds-DNA were eluted with 100 μl/well 0.1 M glycine/0.3 M NaCl pH 2.8 for 20 minutes at room temperature, then neutralised with 2M Tris-HCl pH 7.6 solution. Thereafter, the eluates were concentrated using Amicon Ultra-0.5 centrifugal filter devices from Merck Millipore (Darmstadt, Germany) following the manufacturer’s recommendations. To assess the anti-dsDNA activity of these samples, 1:100 diluted samples were tested using the same commercial kit utilized for anti-dsDNA isolation (INOVA Diagnostics) according to the manufacturer’s instructions.

Cell transfection

Monocytes purified from healthy donors were plated 24 hours before transfection in 6-well plates with complete medium without antibiotics (Opti-MEM, Life Technologies, Madrid, Spain). Cells were transfected with 100 nmol/L miRNA mimic (Life Technologies, Madrid, Spain) for miR-124a, miR-125a -either separately or in conjunction-, and a non-specific control (scrambled) by using siPORT™ NeoFX™ transfection agent (Life Technologies, Madrid, Spain) following manufacturer’s protocols. Forty-two hours after transfection cells were activated with 10 mg/mL LPS (Sigma-Aldrich, Madrid, Spain) for 6h, and potential targets were analyzed by qRT-PCR, Flow cytometry, Flowcytomix, and Western blot. Data were expressed as changes relative to the values of the cells transfected with scrambled control, and set as 100%.

Supplementary Tables

Supplementary Table SI. Parameters related to inflammation and oxidative stress in APS and SLE patients

	Healthy donors (N=56)	APS patients (N=23)	P#	SLE patients (N=64)	*P#*
**INFLAMMATORY PARAMETERS***					
**Tissue Factor** (%)	15.54±11.67	33.51±30.51	0.030	24.87±20.66	0.011
**PAR2 (%)**	5.34±6.24	15.31±21.39	0.007	13.31±20.02	0.020
**sCD40L**(ng/mL)	16.06±15.57	18.10±15.64	n.s.	11.63±11.37	n.s.
**IFN-α** (pg/mL)	119.44±245.55	53.19±150.44	n.s	109.47±171.62	n.s.
**IFN-γ** (pg/mL)	4.72±11.42	0	n.s.	0.39±1.09	n.s.
**IL-1ß** (pg/mL)	12.98±21.26	9.38±16.25	n.s	18.05±34.05	n.s.
**IL-2** (pg/mL)	26.97±38.44	0	n.s.	184.80±290.46	n.s.
**IL-6** (pg/mL)	0.81±1.39	1.87±2.56	n.s.	0.48±0.91	0.001
**IL-8** (pg/mL)	4.06±1.80	6.48±2.80	0.024	27.38±45.12	0.001
**IL-10** (pg/mL)	23.36±65.16	29.31±77.54	n.s.	0.93±1.43	n.s.
**IL-17** (pg/mL)	4.78±15.84	--	--	53.51±91.89	0.024
**IL-23** (pg/mL)	0	--	--	44.48±83.63	0.021
**MCP-1** (pg/mL)	59.81±18.08	96.76±72.44	0.040	192.28±195.99	0.001
**MIP1-α** (pg/mL)	249.93±343.70	463.19±437.91	n.s.	353.01±503.34	n.s.
**sP-Sel** (pg/mL)	196.15±75.32	188.99±67.06	n.s.	158.68±50.21	n.s.
**TNF-α** (pg/mL)	0	0	--	0.30±0.68	n.s.
**t-PA** (ng/mL)	1,79±0.63	2,60±1,49	0.023	3,83±2,36	0.001
**VEGF-A** (pg/mL)	95.18±55.16	101.66±107.19	n.s.	100.38±78.28	n.s.
**OXIDATIVE STATUS***					
**Peroxides/Peroxinytrites** (MIF)$					
Monocytes					
Neutrophils	36.62±12.39	61.17±32.17	0.031	48.21±27.55	0.025
**Mitochondrial membrane**	10.62±5.92	13.69±7.81	0.048	13.70±6.53	0.049
**potential** (% positive cells)					
Monocytes					
Neutrophils	4.62±6.10	20.61±21.74	0.022	12.35±13.05	0.001
**Manganese-SOD**	3.18±7.13	11.22±17.69	0.019	14.07±21.15	0.002
(U/min/mL/µg prot)					
Monocytes					
Neutrophils	0.08±0.05	0.30±0.20	0.001	0.25±0.20	0.042
**Catalase**	2.41±1.13	2.10±0.62	n.s.	2.03±1.13	n.s.
(nmol/min/mL/µg protein)					
Monocytes					
Neutrophils	217.49±103.27	98.68±42.27	0.019	217.62±146.08	n.s.
**GPx**	571.40±404.42	297.18±101.11	0.001	386.28±188.62	0.033
(nmol/min/mL/µg protein)					
Monocytes					
Neutrophils	2.10±0.77	1.13±0.87	0.049	1.89±0.34	n.s.
	0.50±0.19	0.26±0.15	0.001	0.33±0.19	0.004

* Values are mean ± SD

#P<0.05 vs healthy donors

$Unless specified, data are referred to monocytes.

Supplementary TableS II. Correlations/Associations among microRNAs, oxidative stress, inflammation and autoimmunity parameters in APS patients.

APS PATIENTS				
Neutrophils	**OXIDATIVE STRESS***	**INFLAMMATION***	**AUTOIMMUNITY#**	
**Hsa-miR-124a**	**Catalase**<br>0.460/0.009			**ACA-IgG positivity** *(P=0.015)*<br>Neg. 87.85±182.08<br>Pos. 6.58±10.73
**Hsa-miR-125a**	**Catalase**<br>0.494/0.004		**LA positivity** *(P=0.004)*<br>Neg. 95.03±138.70<br>Pos. 16.55±22.42	
**Hsa-miR-125b**	**Catalase**<br>0.544/0.001	**CRP**<br>-0.315/0.024	**LA positivity** *(P=0.002)*<br>Neg. 95.68±122.49<br>Pos. 19.93±23.46	
**Hsa-miR-146a**	**Catalase**<br>0.384/0.030	**CRP**<br>-0.323/0.021	**LA positivity** *(P=0.004)*<br>Neg. 95.65±81.74<br>Pos. 24.59±33.14	
**Hsa-miR-155**	**Catalase**<br>0.525/0.002	**CRP**<br>-0.460/0.001	**LA positivity** *(P=0.001)*<br>Neg. 96.08±68.60<br>Pos. 23.55±23.18	
**Hsa-miR-222**	**Catalase**<br>0.464/0.007	**IL-8**<br>-0.480/0.020		**ACA-IgG positivity** *(P=0.006)*<br>Neg. 85.99±101.17<br>Pos. 27.61±18.45
Monocytes	**OXIDATIVE STRESS***	**INFLAMMATION***	**AUTOIMMUNITY#**	
**Hsa-miR-124a**			**LA positivity (P=0.004)**<br>Neg. 95.03±138.70<br>Pos. 16.55±22.42	
**Hsa-miR-125a**	**ΔΨm**<br>-0.466/0.014	**CRP**<br>-0.287/0.045<br>**TF**<br>-0.449/0.008<br>**PAR-2**<br>-0.507/0.002		**ACA-IgG positivity (P=0.001)**<br>Neg. 84.30±109.08<br>Pos. 5.06±3.53
**Hsa-miR-146a**	**SODmit**<br>0.733/0.016	**IL-8**<br>0.423/0.031		
**Hsa-miR-155**	**ΔΨm**<br>0.459/0.014<br>**GPx**<br>-0.580/0.048	**CRP**<br>0.432/0.002<br>**IL-8**<br>0.468/0.021		

CRP: C-reactive protein; TF: tissue factor expression; PAR2: Protease activated receptor type 2; SODmit: superoxide dismutase mitochondrial; ΔΨm: mitochondrial membrane potential; GPx: glutathione peroxidase.
*Correlation studies: Data are expressed as Spearman Rho coeficient / P value
#Association studies: Data are expressed as the mean ± SD. *P* value in brackets.

Supplementary TableS III. Correlations/Associations among microRNAs, oxidative stress, inflammation and autoimmunity parameters in SLE patients.

SLE PATIENTS				
Neutrophils	**OXIDATIVE STRESS***	**INFLAMMATION***	**AUTOIMMUNITY#**	
**Hsa-miR-124a**	**Catalase (**0.340/0.046)	**IL-23** (-0.435/0.049)	**Anti-dsDNA positivity**<br>(*P= 0.001)*<br>Neg. 69.84±155.66<br>Pos. 2.41±2.41	**ACA-IgG positivity**<br>*(P=0.007)*<br>Neg. 75.83±161.47<br>Pos. 7.06±11.17
**Hsa-miR-125a**	**Peroxides** (-0.313/0.025)<br>**ΔΨm** (-0.292/0.049)	**IL-6** (-0.519/0.001)<br>**IL-8** (-0.369/0.029)		**ACA-IgG positivity**<br>*(P=0.001)*<br>Neg. 81.41±77.08<br>Pos. 23.16±25.39
**Hsa-miR-125b**	**Catalase (**0.420/0.011)	**IL-23 (**-0.441/0.040)		
**Hsa-miR-146a**		**t-PA (**-0.373/0.027)	**Anti-dsDNA positivity**<br>(*P= 0.002)*<br>Neg. 85.15±82.83<br>Pos. 24.77±21.07	
**Hsa-miR-155**	**Catalase** (0.403/0.015)<br>**Peroxides (**-0.280/0.046)	**CRP (**-0.341/0.005)<br>**IL-6 (**-0.453/0.005)<br>**IL-8 (**-0.371/0.026)<br>**t-PA (**-0.322/0.049)		**ACA-IgG positivity**<br>*(P=0.043)*<br>Neg. 93.62±80.48<br>Pos. 42.96±45.63
**Hsa-miR-222**	**Catalase (**0.356/0.033)	**IL-6 (-**0.388/0.019)<br>**IL-17 (**-0.375/0.048)<br>**IL-23 (**-0.423/0.044)	**Anti-dsDNA positivity**<br>(*P= 0.003)*<br>Neg. 77.03±92.55<br>Pos. 27.61±19.81	**ACA-IgG positivity**<br>*(P=0.003)*<br>Neg. 78.74±93.50<br>Pos. 26.56±24.51
Monocytes	**OXIDATIVE STRESS***	**INFLAMMATION***	**AUTOIMMUNITY#**	
**Hsa-miR-125a**		**CRP (**-0.281/0.027)<br>**TF (**-0.540/0.001)<br>**PAR-2 (**-0.493/0.001)		
**Hsa-miR-146a**		**IL-17 (**0.644/0.049)	**Anti-dsDNA positivity**<br>Neg. 123.20±129.03<br>Pos. 259.37±212.18<br>(*P= 0.039)*	**ACA-IgG positivity**<br>Neg. 128.86±131.81<br>Pos. 300.77±200.45<br>*(P=0.009)*
**Hsa-miR-155**	**ΔΨm (**0.342/0.020)	**CRP (**0.315/0.013)<br>**IL-23 (**0.649/0.042)	**Anti-dsDNA positivity**<br>Neg. 133.02±126.44<br>Pos. 263.07±216.88<br>(*P= 0.046)*	**ACA-IgG positivity**<br>Neg. 146.13±144.42<br>Pos. 299.24±227.89<br>*(P=0.034)*

CRP: C-reactive protein; TF: tissue factor expression; PAR2: Protease activated receptor type 2; SODmit: superoxide dismutase mitochondrial; ΔΨm: mitochondrial membrane potential; GPx: glutathione peroxidase.
*Correlation studies: Data are expressed as Spearman Rho coeficient / P value
#Association studies: Data are expressed as the mean ± SD. *P* value in brackets.

Individual treatments in primary APS patients

ASA: Aspirin; HCQ: hydroxychloroquine; the hyphens denote not available information; NA: not applicable.

Patient	Age (years)	Sex	Arterial Thrombosis	Venous Thrombosis	Pregnancy morbidity	Carotid Intimate Media thickness	Anticoagulants/ Antiplatelets	Antimalarials (HCQ)	Corticosteroids (Prednisone)
1	46	M	YES	NO	-	Pathological	ASA	YES	NO
2	35	F	NO	YES	YES	Normal	ASA	NO	NO
3	57	F	YES	NO	NO	Normal	Acenocoumarol	NO	NO
4	36	F	NO	NO	YES	Normal	NO	NO	NO
5	61	M	YES	NO	NA	Pathological	Acenocoumarol	NO	NO
6	34	F	YES	NO	NO	Normal	Acenocoumarol	YES	NO
7	37	F	NO	NO	YES	Normal	ASA	NO	NO
8	38	F	NO	NO	YES	Normal	ASA	NO	NO
9	71	M	YES	YES	NA	Pathological	ASA	NO	NO
10	49	F	YES	NO	NO	Pathological	ASA	NO	NO
11	57	F	YES	YES	YES	Normal	Acenocoumarol	YES	YES
12	70	F	YES	NO	NO	Pathological	Acenocoumarol	NO	NO
13	36	F	YES	NO	YES	-	Acenocoumarol	NO	NO
14	35	F	YES	NO	NO	Normal	ASA	NO	NO
15	32	M	YES	NO	NA	Normal	ASA	NO	NO
16	36	M	YES	NO	NA	Normal	ASA	NO	NO
17	40	F	NO	YES	YES	Normal	Acenocoumarol	YES	YES
18	50	M	YES	NO	NA	Pathological	Acenocoumarol	NO	NO
19	56	F	YES	NO	NO	Pathological	Acenocoumarol	NO	NO
20	41	F	NO	NO	YES	Normal	ASA	NO	NO
21	44	F	YES	NO	YES	Normal	Acenocoumarol	NO	NO
22	73	F	NO	YES	NO	Pathological	ASA	YES	NO
23	48	F	YES	NO	NO	Pathological	Acenocoumarol	NO	NO

Individual treatments in SLE patients positive for aPL

Patient	Age (years)	Sex	Arterial Thrombosis	Venous Thrombosis	Pregnancy morbidity	Carotid Intimate media thickness	Anticoagulants/ Antiplatelets	Antimalarials (HCQ)	Immunosuppresant drugs	Corticosteroids (Prednisone)
1	39	M	NO	YES	NA	Normal	Acenocoumarol	YES	NO	NO
2	53	F	NO	YES	YES	Pathological	ASA	YES	NO	YES
3	45	F	YES	NO	NO	Normal	Acenocoumarol	NO	NO	YES
4	31	F	YES	NO	YES	Normal	ASA	YES	NO	YES
5	32	F	NO	YES	NO	Normal	ASA	YES	NO	YES
6	54	F	YES	YES	YES	Normal	Acenocoumarol	NO	NO	YES
7	47	F	YES	NO	NO	Pathological	Acenocoumarol	YES	YES	YES
8	71	F	YES	YES	NO	Pathological	Acenocoumarol	YES	NO	YES
9	48	M	YES	NO	NA	Pathological	ASA	YES	YES	YES
10	35	F	YES	NO	YES	-	Acenocoumarol	YES	NO	YES
11	41	F	YES	YES	NO	Normal	ASA	YES	NO	YES
12	57	F	YES	NO	NO	Pathological	Acenocoumarol	NO	NO	YES
13	41	F	NO	NO	NO	Normal	ASA	YES	YES	YES
14	59	F	YES	YES	YES	Pathological	Acenocoumarol	NO	NO	YES
15	48	F	YES	NO	NO	Pathological	Acenocoumarol	NO	NO	NO
16	36	F	YES	NO	NO	Normal	ASA	YES	NO	YES
17	52	F	NO	YES	NO	Pathological	Acenocoumarol	YES	NO	YES
18	43	M	NO	YES	NA	Normal	Acenocoumarol	YES	NO	YES
19	49	F	YES	NO	YES	Pathological	ASA	YES	NO	YES
20	53	F	YES	NO	NO	-	Acenocoumarol	NO	NO	NO
21	54	F	YES	NO	YES	Normal	ASA	YES	NO	YES
22	40	F	YES	NO	NO	Normal	Acenocoumarol	YES	NO	YES
23	51	F	YES	NO	NO	Pathological	Acenocoumarol	NO	NO	YES

ASA: Aspirin; HCQ: hydroxychloroquine; the hyphens denote not available information; NA: not applicable.

Individual treatments in SLE patients negative for aPL

Patient	Age (years)	Sex	Arterial Thrombosis	Venous Thrombosis	Pregnancy morbidity	CarotidIntimate Media thickness	Anticoagulants/ Antiplatelets	Antimalarials (HCQ)	Corticosteroids (Prednisone)	Immunosuppressant drugs
1	27	F	YES	NO	NO	Normal	Acenocoumarol	YES	YES	YES
2	43	F	NO	NO	NO	Normal	ASA	YES	YES	NO
3	32	F	NO	NO	NO	Normal	ASA	YES	YES	NO
4	51	F	YES	NO	NO	Normal	ASA	YES	YES	NO
5	53	F	YES	NO	NO	Normal	ASA	YES	YES	NO
6	73	F	NO	NO	NO	Pathological	Acenocoumarol	YES	YES	NO
7	29	F	NO	NO	NO	Normal	ASA	YES	YES	NO
8	30	M	NO	NO	NA	Normal	NO	NO	YES	SI
9	45	F	NO	NO	NO	Normal	ASA	YES	YES	NO
10	45	F	NO	NO	NO	Normal	ASA	YES	YES	NO
11	32	F	NO	NO	NO	Normal	ASA	YES	YES	NO
12	40	M	NO	NO	NA	Normal	NO	YES	YES	NO
13	42	F	NO	NO	NO	Pathological	ASA	YES	YES	NO
14	31	F	NO	NO	NO	Normal	NO	NO	NO	NO
15	45	F	NO	NO	NO	Pathological	NO	YES	NO	NO
16	41	F	NO	NO	NO	-	ASA	YES	YES	YES
17	20	F	NO	NO	NO	Normal	ASA	YES	YES	YES
18	50	F	NO	NO	NO	Normal	ASA	YES	YES	NO
19	36	F	NO	NO	NO	-	NO	YES	YES	NO
20	45	F	NO	NO	NO	Normal	NO	YES	NO	NO
21	29	F	NO	NO	NO	Normal	ASA	YES	YES	NO
22	28	F	NO	NO	NO	-	ASA	YES	YES	NO
23	25	F	NO	NO	NO	Normal	ASA	YES	NO	NO
24	60	F	NO	NO	NO	Pathological	ASA	YES	YES	NO
25	35	F	NO	NO	NO	Normal	NO	YES	YES	YES
26	29	F	NO	NO	NO	Normal	ASA	YES	YES	YES
27	57	M	YES	NO	NA	Pathological	ASA	YES	NO	NO
28	29	M	NO	NO	NA	Normal	NO	YES	YES	YES
29	46	F	NO	NO	NO	Normal	NO	YES	YES	YES
30	37	F	NO	NO	NO	Normal	ASA	YES	YES	YES
31	27	F	NO	NO	NO	-	ASA	YES	YES	YES

32	41	F	NO	NO	NO	Normal	ASA	YES	YES	NO
33	26	F	NO	NO	NO	Normal	NO	YES	YES	NO
34	53	F	NO	NO	NO	Normal	ASA	YES	YES	YES
35	64	F	NO	NO	NO	Pathological	ASA	YES	YES	YES
36	35	F	NO	NO	NO	Normal	ASA	YES	YES	NO
37	18	M	NO	NO	NA	Normal	NO	NO	NO	NO
38	24	F	NO	NO	NO	Normal	NO	YES	YES	NO
39	28	F	NO	NO	NO	Normal	ASA	YES	YES	NO
40	48	F	NO	NO	NO	Pathological	ASA	YES	YES	YES
41	35	F	NO	NO	NO	Normal	ASA	YES	YES	NO

ASA: Aspirin; HCQ: hydroxychloroquine; the hyphens denote not available information; NA: not applicable.

<u>**Supplementary Figures**</u>

Supplementary Figure S1

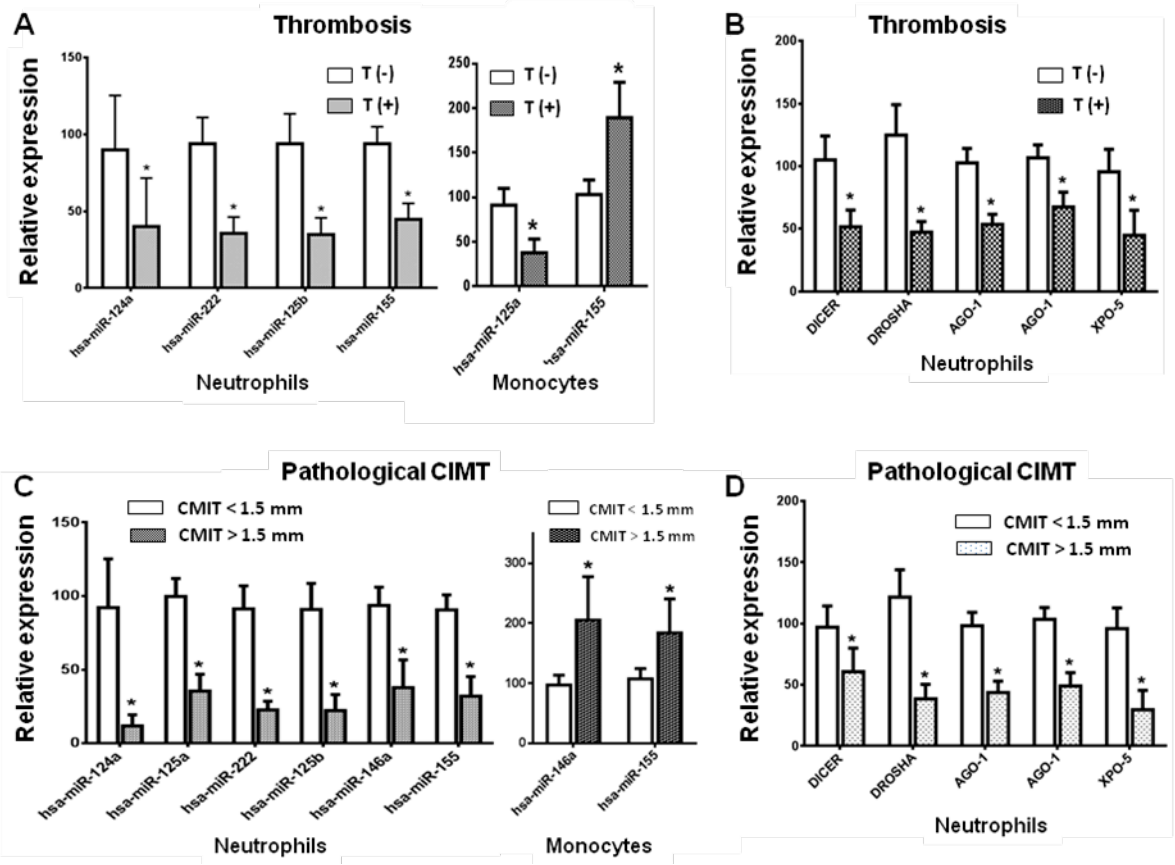

Supplementary Figure S1. Association studies among miRNAs or biomarkers of miRNA biogenesis expression levels and increased carotid intima-media thickness (CIMT) or occurrence of thrombotic events (T) in SLE patients. (A) Relationship between the occurrence of thrombotic events and the expression of the selected miRNAs in neutrophils and monocytes. (B) Relationship between the occurrence of thrombotic events and the expression of various biomarkers of miRNA biogenesis in neutrophils. (C) Relationship between the presence of increased CIMT and the expression of the selected miRNAs in neutrophils and monocytes. (D) Relationship between the presence of increased CIMT and the expression of various biomarkers of miRNA biogenesis in neutrophils. Significant differences versus patients without thrombosis or versus patients without increased CIMT (*P<0.05).

Supplementary Figure S2

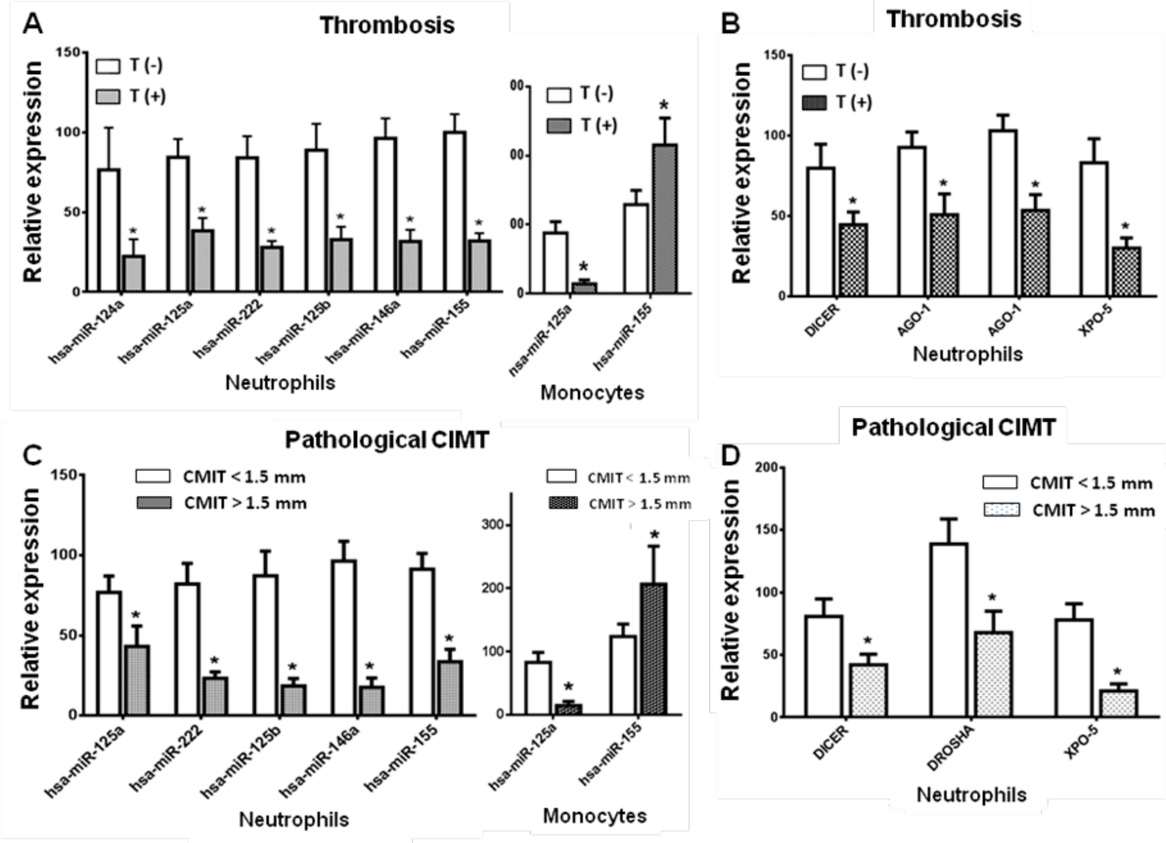

Supplementary Figure S2. Association studies among miRNAs or biomarkers of miRNA biogenesis expression levels and increased carotid intima-media thickness (CIMT) or occurrence of thrombotic events (T) in primary APS patients. (A) Relationship between the occurrence of thrombotic events and the expression of the selected miRNAs in neutrophils and monocytes. (B) Relationship between the occurrence of thrombotic events and the expression of various biomarkers of miRNA biogenesis in neutrophils. (C) Relationship between the presence of increased CIMT and the expression of the selected miRNAs in neutrophils and monocytes. (D) Relationship between the presence of increased CIMT and the expression of various biomarkers of miRNA biogenesis in neutrophils. Significant differences versus patients without thrombosis or versus patients without increased CIMT (*P<0.05).

Supplementary Figure S3

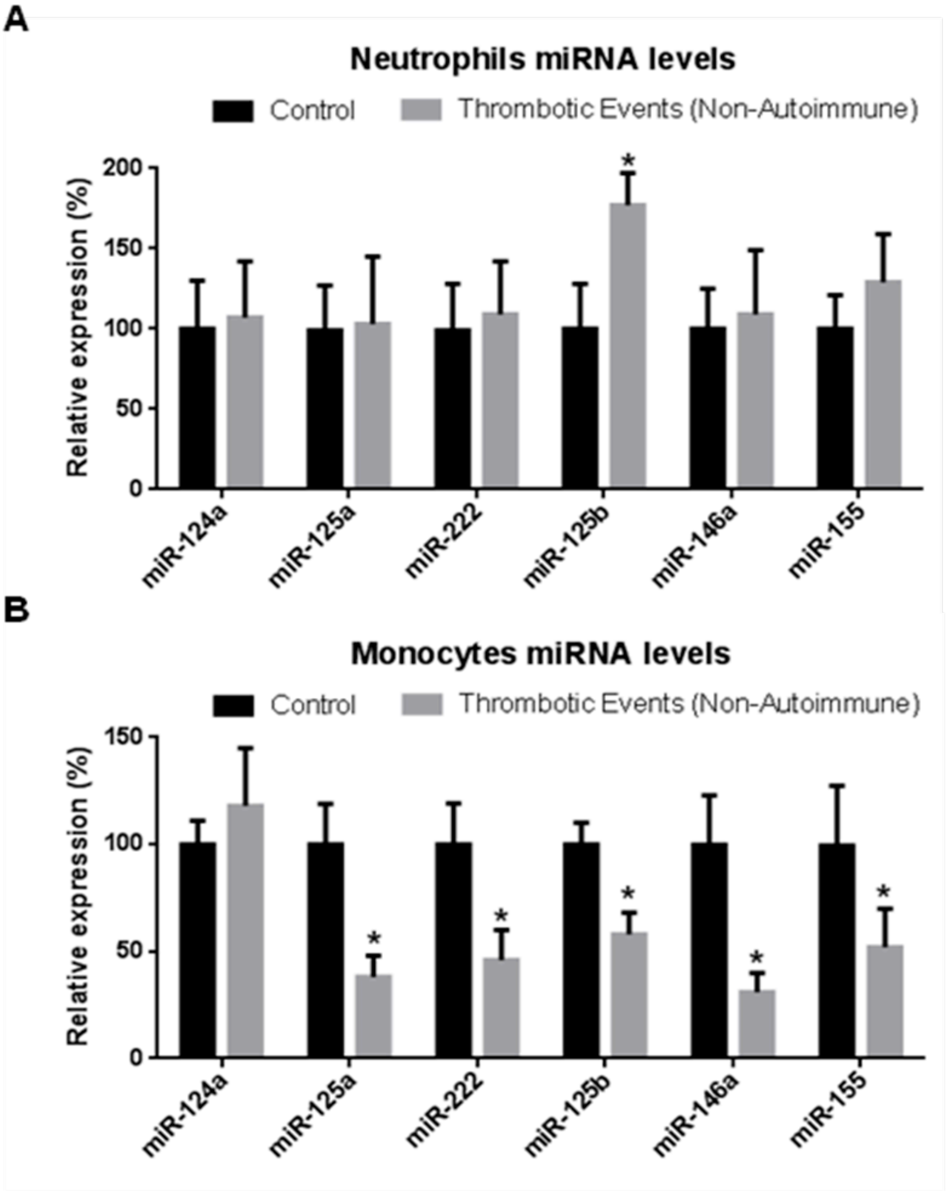

Supplementary Figure S3. Expression levels of the selected miRNAs in neutrophils and monocytes from patients with thrombosis, but without aPL.

miRNA levels were measured in all the subjects included in the study (20 patients with previous venous or arterial thrombosis events and 20 healthy donors) on isolated neutrophils (A) or monocytes (B) by qRT-PCR and normalized with U6 snRNA. Differences were analyzed by Student's t test. Statiscal significance was taken as $p<0.05$.

Supplementary Figure S4

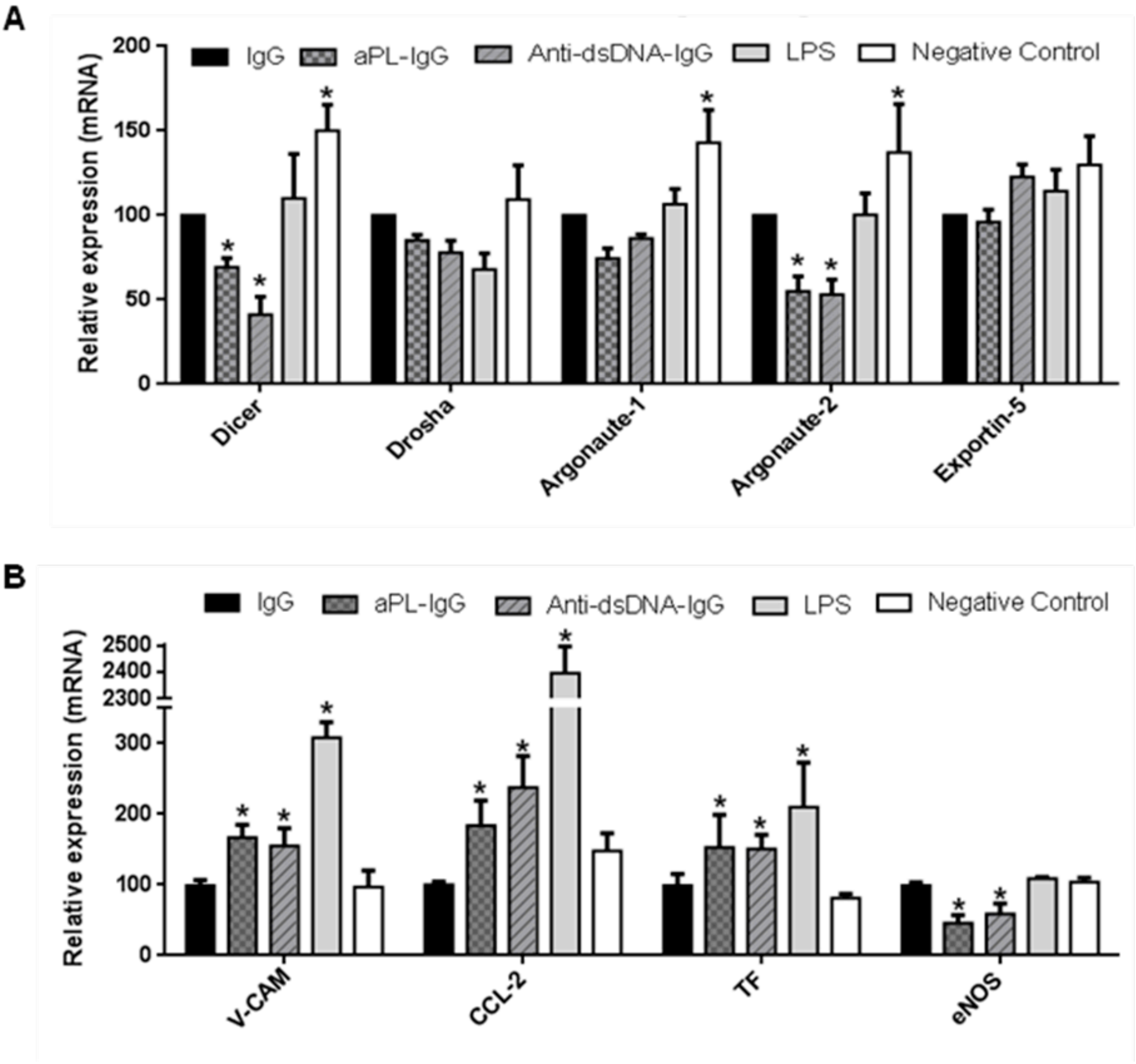

Supplementary Figure S4. In vitro effects of aPL-IgG and anti-dsDNA-IgG antibodies on the expression of proteins of biogenesis and inflammatory markers.

Endothelial cells were treated in vitro with aPL-IgG antibodies purified from APS patients' serum, or with anti-dsDNA-IgG antibodies purified from SLE patients' serum, or with LPS, or with culture medium, or with synthetic human IgG. (A) Relative mRNA expression levels of miRNA biogenesis proteins, including Dicer, Drosha, Ago1, Ago2 and Exportin-5. Values are the mean and SEM from 4 independent experiments. Significant differences (*P<0.05) vs ECs treated with synthetic IgG. (B) Relative mRNA expression levels of various inflammatory mediators after the above described

treatments. Values are the mean and SEM from 4 independent experiments. Significant differences (*P<0.05) vs ECs treated with synthetic IgG.

Supplementary Figure S5

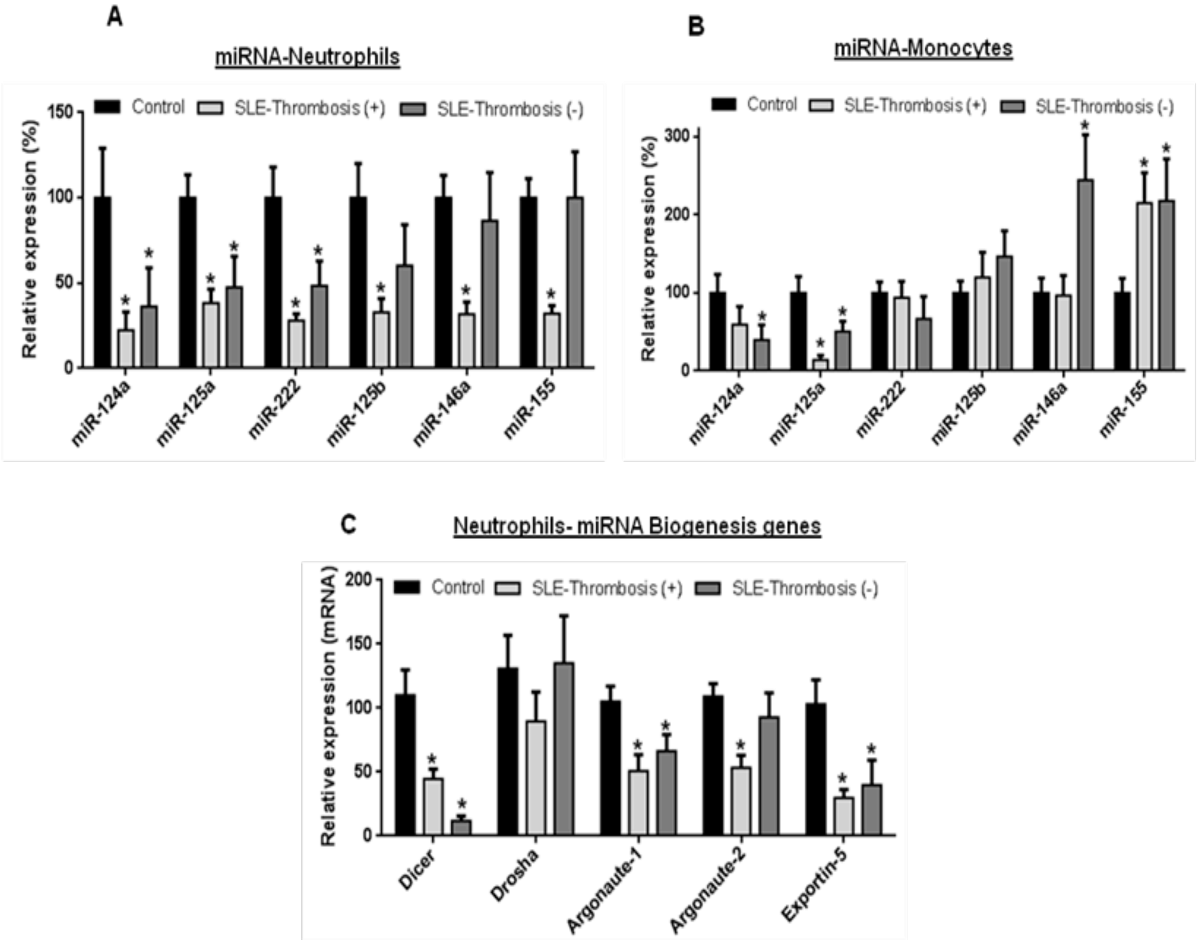

Supplementary Figure S5. Expression levels of the selected miRNAs and biomarkers of miRNA-biogenesis in neutrophils and monocytes from SLE patients with thrombosis or without thrombosis in relation to healthy donors. (A) miRNA levels were measured in monocytes and neutrophils isolated from SLE patients with or without previous thrombotic events and in healthy donors by qRT-PCR and normalized with U6 snRNA. (B) Relative mRNA expression levels of miRNA biogenesis proteins, including Dicer, Drosha, Ago1, Ago2 and Exportin-5. Differences were analyzed by Student’s t test. Statistical significance was taken as p<0.05.

Supplementary Figure S6

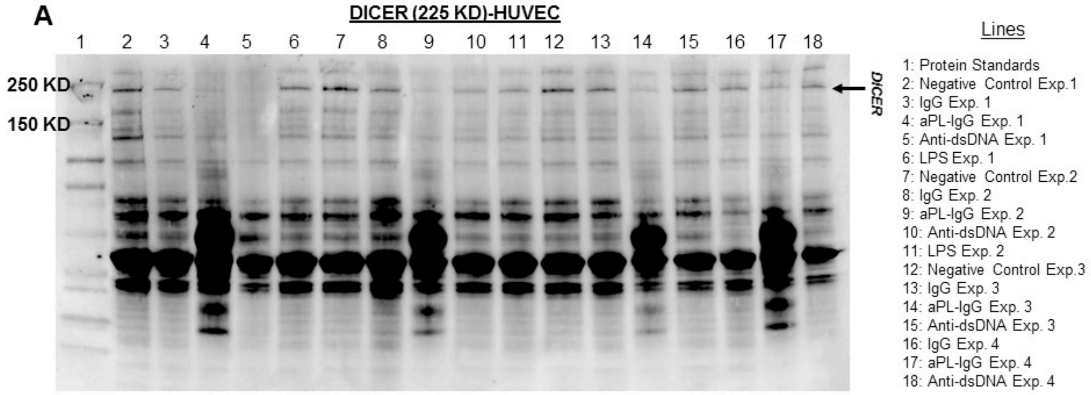

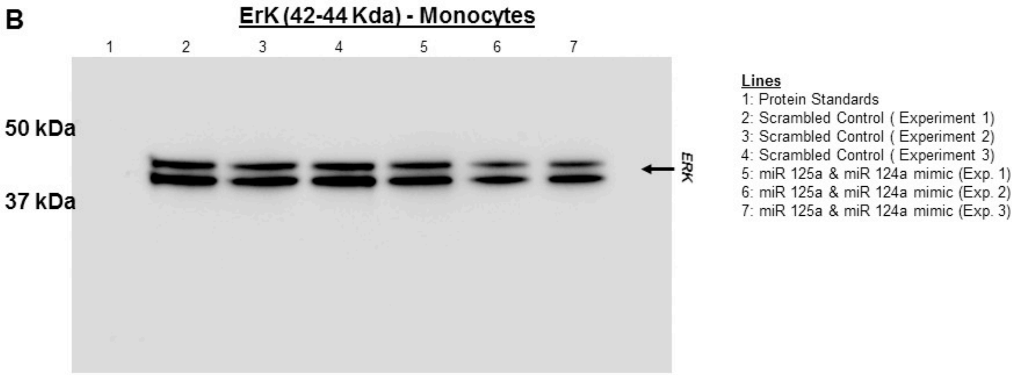

Supplementary Figure 6. Full-length blots for key data. (A) Western blot of samples from independent experiments showing Dicer expression after the treatments indicated in ECs.

(B) Western blot of samples from 3 independent experiments showing the changes promoted in ERK expression by the simultaneous transfection of monocytes with miR-124a and miR-125a mimics.
